# Molecular mechanisms underlying metamorphosis in the most-ancestral winged insect

**DOI:** 10.1073/pnas.2114773119

**Published:** 2022-02-25

**Authors:** Genta Okude, Minoru Moriyama, Ryouka Kawahara-Miki, Shunsuke Yajima, Takema Fukatsu, Ryo Futahashi

**Affiliations:** ^a^Department of Biological Sciences, Graduate School of Science, The University of Tokyo, Tokyo 113-0033, Japan;; ^b^Bioproduction Research Institute, National Institute of Advanced Industrial Science and Technology, Tsukuba 305-8566, Japan;; ^c^NODAI Genome Research Center, Tokyo University of Agriculture, Tokyo 156-8502, Japan;; ^d^Department of Bioscience, Tokyo University of Agriculture, Tokyo 156-8502, Japan;; ^e^Graduate School of Life and Environmental Sciences, University of Tsukuba, Tsukuba 305-8572, Japan

**Keywords:** metamorphosis, damselfly, dragonfly, Odonata, MEKRE93 pathway

## Abstract

Insects comprise over half of the described species, and the acquisition of metamorphosis must have contributed to their diversity and prosperity. The order Odonata (dragonflies and damselflies) is among the most-ancestral insects with drastic morphological changes upon metamorphosis, in which understanding of the molecular mechanisms will provide insight into the evolution of incomplete and complete metamorphosis in insects. In order to identify metamorphosis-related genes in Odonata, we performed comprehensive RNA-sequencing of the blue-tailed damselfly *Ischnura senegalensis* at different developmental stages. Comparative RNA-sequencing analyses between nymphs and adults identified eight nymph-specific and seven adult-specific transcripts. RNA interference (RNAi) of these candidate genes demonstrated that three transcription factors, *Krüppel homolog 1* (*Kr-h1*), *broad*, and *E93* play important roles in metamorphosis of both *I. senegalensis* and a phylogenetically distant dragonfly, *Pseudothemis zonata*. *E93* is essential for adult morphogenesis, and RNAi of *Kr-h1* induced precocious metamorphosis in epidermis via up-regulation of *E93*. Precocious metamorphosis was also induced by RNAi of the juvenile hormone receptor *Methoprene-tolerant* (*Met*), confirming that the regulation of metamorphosis by the MEKRE93 (Met-Kr-h1-E93) pathway is conserved across diverse insects including the basal insect lineage Odonata. Notably, RNAi of *broad* produced unique grayish pigmentation on the nymphal abdominal epidermis. Survey of downstream genes for *Kr-h1*, *broad*, and *E93* uncovered that unlike other insects, *broad* regulates a substantial number of nymph-specific and adult-specific genes independently of *Kr-h1* and *E93*. These findings highlight the importance of functional changes and rewiring of the transcription factors *Kr-h1*, *broad*, and *E93* in the evolution of insect metamorphosis.

## Significance

As caterpillars metamorphose to butterflies, insects change their appearance dramatically through metamorphosis. Some insects have an immobile pupal stage for morphological remodeling (homometaboly). Other insects, such as cockroaches, have no pupal stage, and the juveniles and adults are morphologically similar (hemimetaboly). Notably, among the most-ancestral hemimetabolous insects, dragonflies drastically alter their appearance from aquatic nymphs to aerial adults. In dragonflies, we showed that transcription factors *Kr-h1* and *E93* are essential for regulating metamorphosis as in other insects, while *broad*, the master gene for pupation in holometabolous insects, regulates a number of both nymph-specific genes and adult-specific genes, providing insight into what evolutionary trajectory the key transcription factor *broad* has experienced before ending up with governing pupation and holometaboly.

Insects comprise over half of the described species and are regarded as the most prosperous organismal group in the world (1). Over 98% of the insect species undergo “metamorphosis,” by which they dramatically change their morphology from larvae (nymphs) to adults, with most of them developing wings. Drastic metamorphosis together with the acquisition of wings must have contributed to the remarkable diversity of insects by enabling them to exploit different habitats and resources (2–4).

The genetic and endocrinological mechanisms underlying insect metamorphosis have been elucidated primarily using model insects such as the fruit fly *Drosophila melanogaster* and the silkworm *Bombyx mori*, representatives of holometabolous insects that undergo complete metamorphosis with the pupal stage between larva and adult (5). Two insect hormones, juvenile hormone (JH) and ecdysteroid (20-hydroxyecdysone; 20E), regulate insect metamorphosis, and their effects and mechanisms of action have been studied in diverse insects (5, 6). In general, 20E triggers larva (nymph)-to-larva (nymph) ecdysis in the presence of JH, whereas 20E induces pupal and/or adult metamorphosis in the absence of JH. After the elucidation of the intracellular JH receptor gene *Methoprene-tolerant* (*Met*) (7, 8), the molecular mechanisms involved in metamorphosis have been functionally analyzed in a variety of insects, including hemimetabolous insects that undergo incomplete metamorphosis from nymphs to adults without the pupal stage.

During larva (nymph)-to-larva (nymph) ecdysis, JH binds to its receptor Met, and subsequently, the transcription factor gene *Krüppel homolog 1* (*Kr-h1*) suppresses metamorphosis to pupa or adult (9). By contrast, during larva (nymph)-to-adult metamorphosis, in the absence of JH, another transcription factor gene *E93* induces metamorphosis to adult (10). *Kr-h1* inhibits the expression of *E93* and vice versa, thereby regulating metamorphosis via the cascade called the MEKRE93 (Met-Kr-h1-E93) pathway (11). The function of MEKRE93 pathway is conserved across holometabolous insects (12, 13) and hemimetabolous insects such as cockroaches (Blattodea) (12), crickets (Orthoptera) (14), and true bugs (Hemiptera) (15, 16). A major difference in the regulatory mechanism of metamorphosis between holometabolous and hemimetabolous insects resides in the contribution of another transcription factor *broad* (also known as *broad-complex*) gene. In holometabolous insects, *broad* triggers pupation. In hemimetabolous insects, meanwhile, *broad* is primarily involved in wing and ovipositor development (12, 14, 17–19). It should be noted that in the milkweed bug *Oncopeltus fasciatus* (Hemiptera), in which the function of *broad* was first analyzed among hemimetabolous insects, RNA interference (RNAi) of *broad* inhibits the changes in pigmentation patterns that are specific to each nymphal instar, suggesting that *broad* regulates progressive morphogenesis (18). Three transcription factors, *Kr-h1*, *E93*, and *broad*, have been defined as key factors forming Metamorphic Gene Network (MGN) (20).

**Fig. 1.**
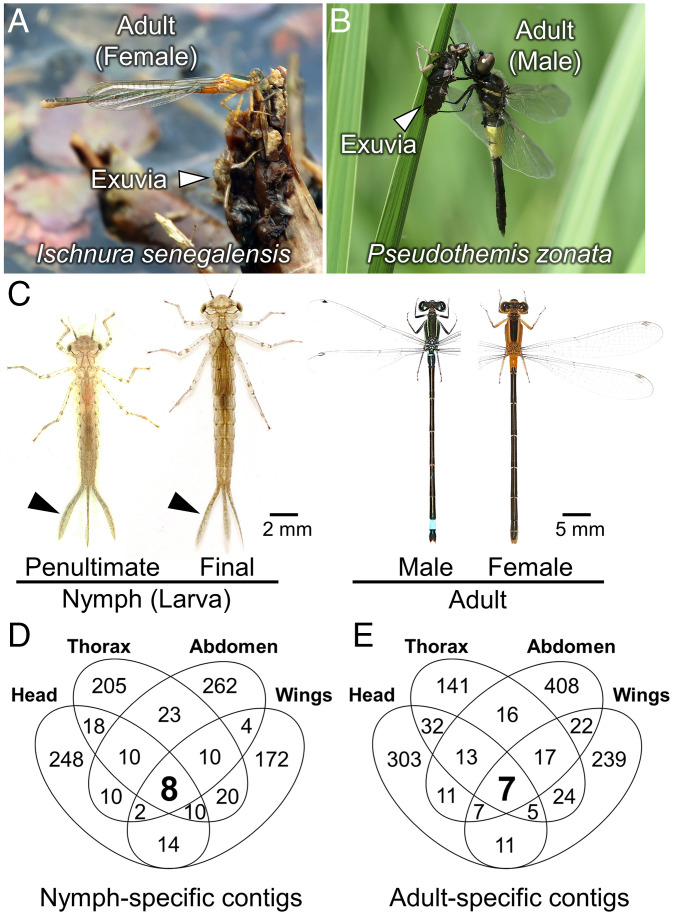
Metamorphosis of Odonata and identification of nymph-specific and adult-specific genes in the blue-tailed damselfly *I. senegalensis* (Coenagrionidae). (*A*) Adult emergence of *I. senegalensis* (Coenagrionidae). (*B*) Adult emergence of the pied skimmer dragonfly *P. zonata* (Libellulidae). Image is courtesy of Hiroyuki Futahashi. (*C*) Nymphs and adults of *I. senegalensis*. Black arrowheads indicate nymph-specific caudal gills. (*D*) The number of nymph-specific contigs identified by the screening criteria in this study. (*E*) The number of adult-specific contigs identified by the screening criteria in this study.

The most-ancestral winged insects with metamorphosis are dragonflies and damselflies (Odonata) (Fig. 1 *A* and *B*) and mayflies (Ephemeroptera), in which functional analysis of the MGN genes should shed light on the origin of metamorphosis (5, 21, 22). In Odonata, the depletion of JH in final nymphal instar is important for nymph-to-adult metamorphosis, because metamorphosis is suppressed by application of JH analog on the first day of final nymphal instar (23), whereas 20E titer increases prior to each ecdysis (24), suggesting that the fluctuation of endogenous JH and 20E titers through postembryonic development in Odonata is similar with that in other hemimetabolous insects (5, 6). However, the molecular mechanisms underlying metamorphosis in Odonata and Ephemeroptera remain largely unknown, mainly due to the lack of molecular genetic tools. We have focused on the blue-tailed damselfly *Ischnura senegalensis* as a model dragonfly species, established a laboratory-rearing system (25), and developed a method for analyzing gene function by electroporation-mediated RNAi (26, 27).

Here we identified eight nymph-specific and seven adult-specific genes by comprehensive RNA-sequencing of *I. senegalensis*. Subsequent electroporation-mediated RNAi of the candidate genes and RNA-sequencing after RNAi experiments demonstrated that the interactions of three transcription factor genes, *Kr-h1*, *broad*, and *E93*, were modified through insect evolution for the regulation of metamorphosis.

## Results

### Screening for Nymph-Specific and Adult-Specific Genes.

First, we investigated the gene-expression profiles by comprehensive RNA-sequencing for the following 82 samples from various developmental stages, body regions, and sexes of *I. senegalensis*: 13 whole-body samples from eggs to seventh instar nymphs (13 developmental stages), 45 samples of penultimate instar and final instar nymphs from head, thorax, abdomen, wing buds, and caudal gills (nine developmental stages), and 24 samples of adults from head, thorax, abdomen, and wings (six individuals consisting of immature and mature males, gynochrome females, and androchrome females) (*SI Appendix*, Fig. S1 and Table S1). Here, we adopt the term “nymph” instead of “larva” to distinguish between holometabolous and hemimetabolous insects, although the majority of previous studies on Odonata used the term “larva,” because dragonflies and damselflies drastically change the external morphology through metamorphosis unlike most other hemimetabolous insects (28, 29).

**Fig. 2.**
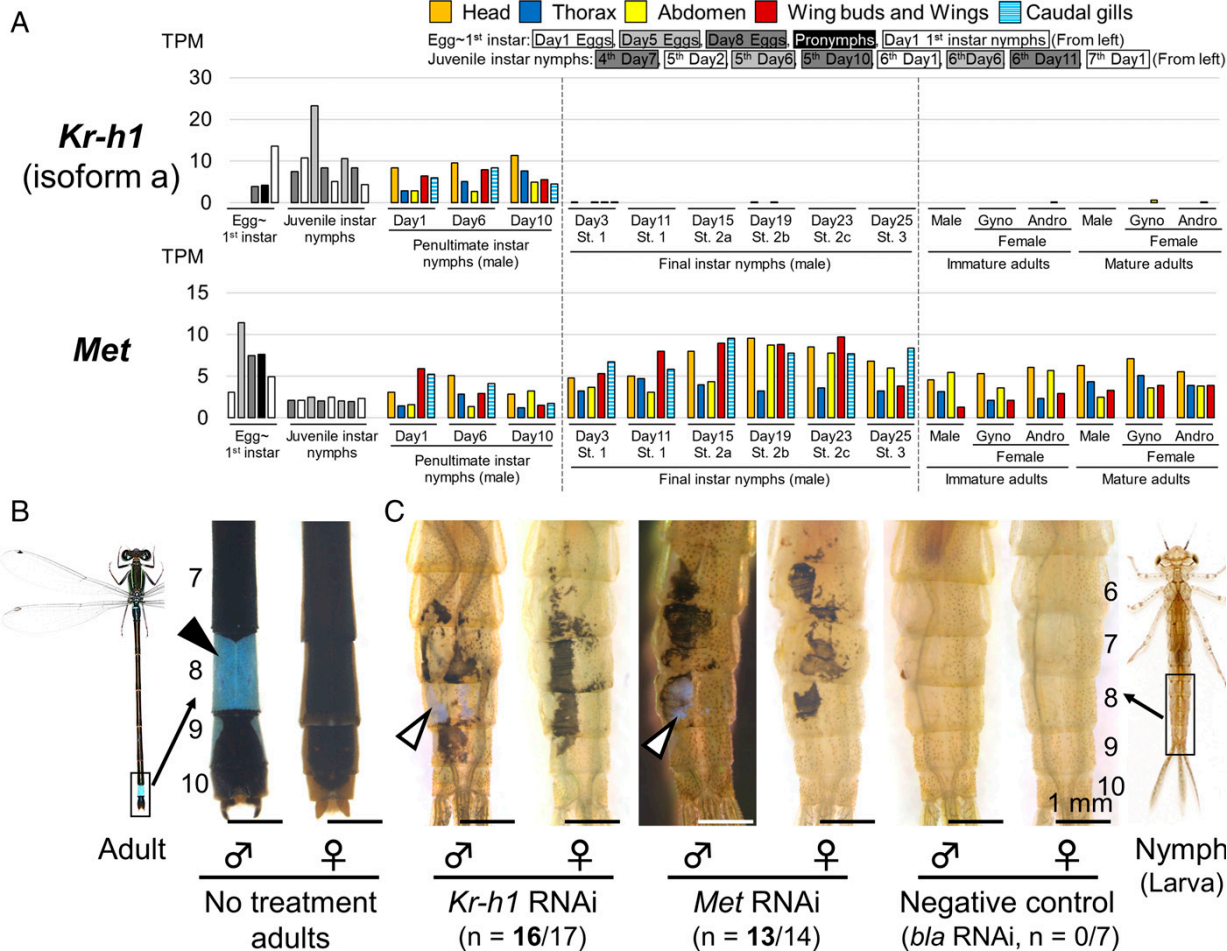
*Kr-h1* and *Met* are essential for maintenance of nymphal status in *I. senegalensis*. (*A*) Expression levels of *Kr-h1* (isoform a) and *Met*. (*B*) Abdominal tip of adults without any treatments. The black arrowhead indicates male-specific, light-blue coloration on the eighth abdominal segment. (*C*) RNAi phenotypes around the dorsal eighth abdominal segment. Numbers of parentheses indicate the number of individuals affected by RNAi/number of molted nymphs. White arrowheads indicate light-blue coloration, which is reminiscent of light-blue markings of adult males. Numbers beside the abdomens indicate the number of abdominal segments.

**Fig. 3.**
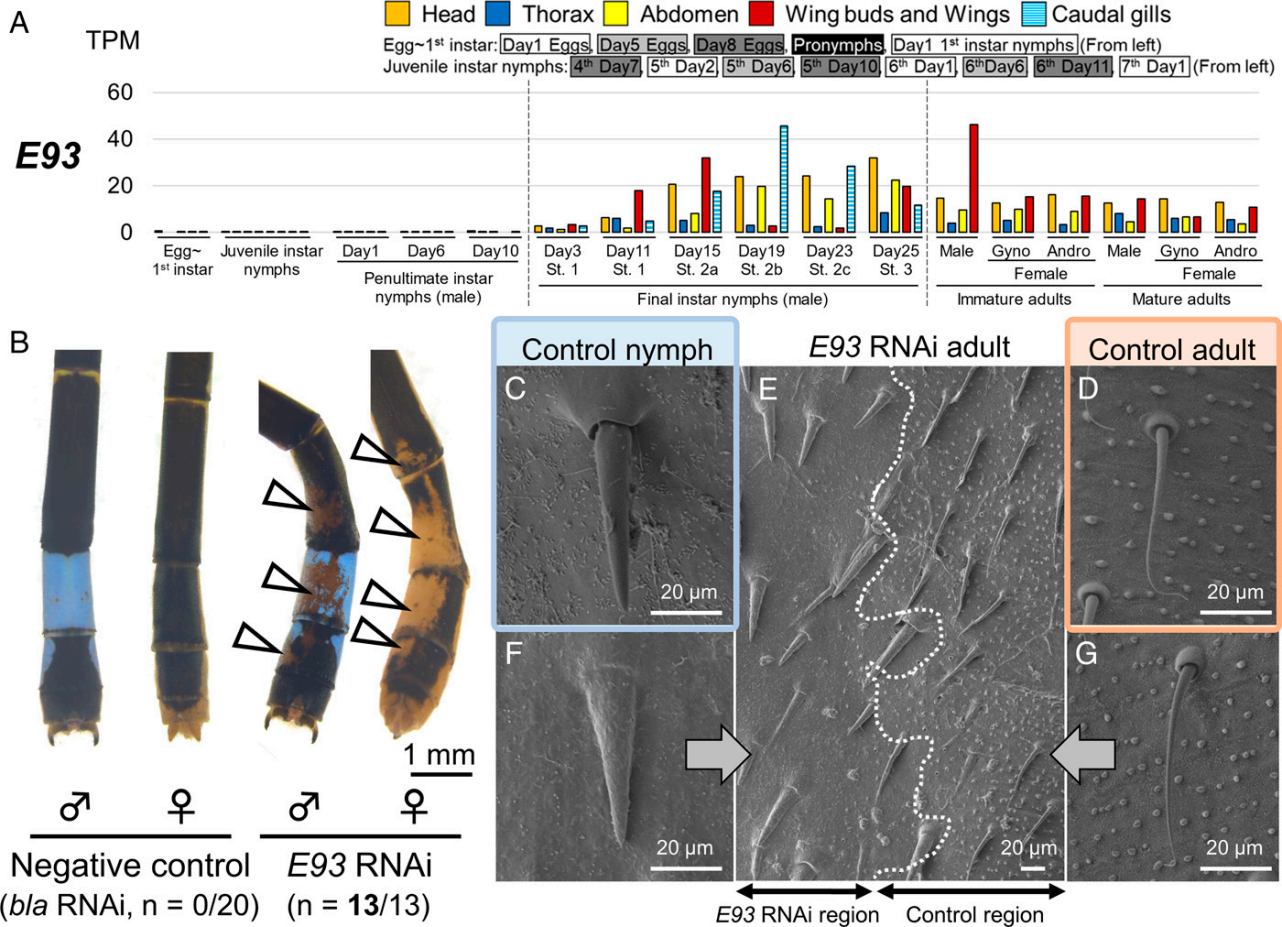
*E93* is essential for adult morphogenesis in *I. senegalensis*. (*A*) Expression levels of *E93*. (*B*) RNAi phenotypes around the dorsal eighth abdominal segment. Numbers of parentheses indicate the number of individuals affected by RNAi/number of emerged adults. White arrowheads indicate the suppression of adult pigmentation. (*C*–*G*) SEM observation on the abdominal surface. (*C*) A nymph without any treatments. (*D*) An adult without any treatments. (*E*) *E93* RNAi individuals. *E93* RNAi region is in the left side of the photo. (*F*) A magnified view of *E93* RNAi region. (*G*) A magnified view of control region of *E93* RNAi individual.

**Fig. 4.**
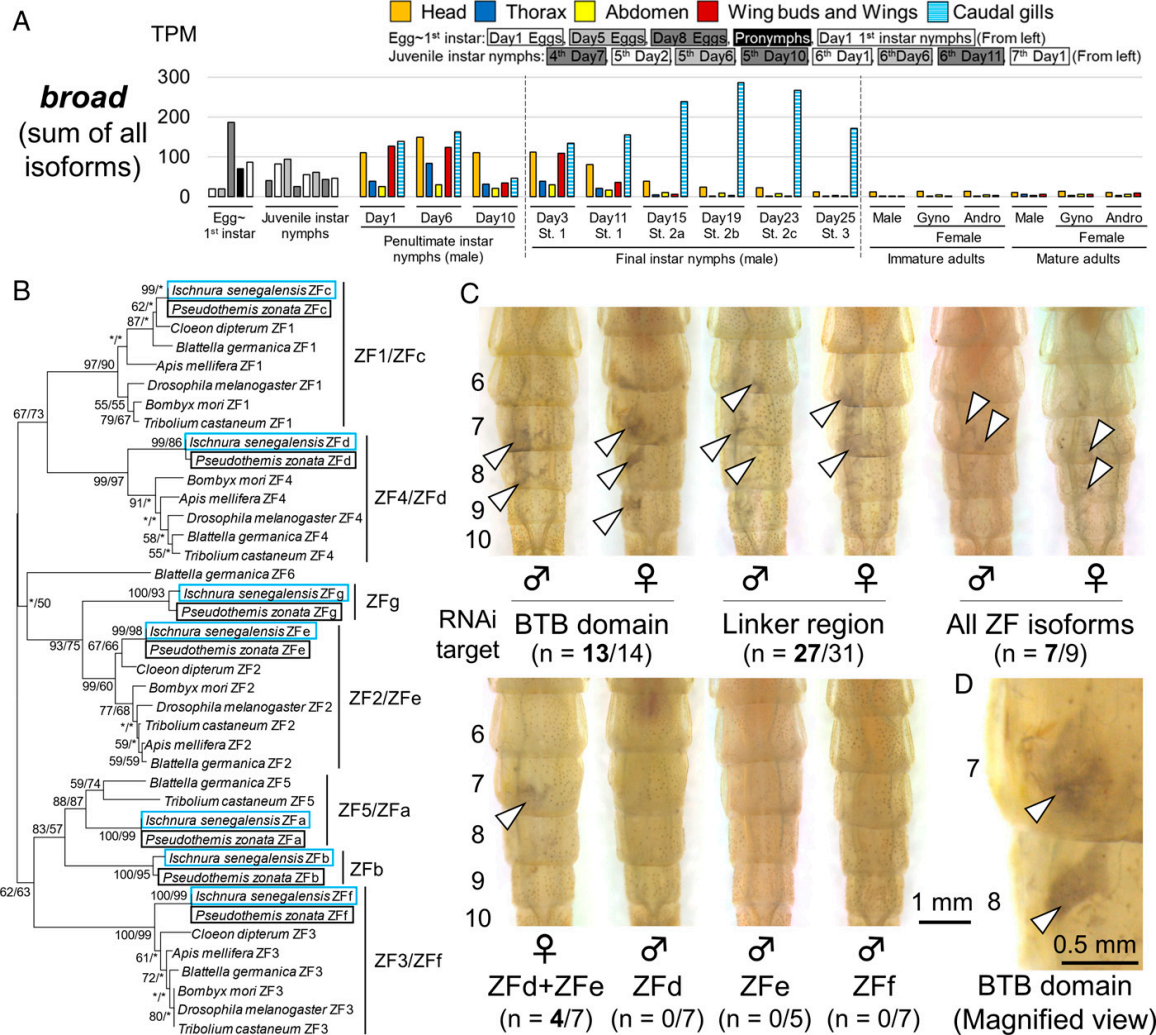
*broad* RNAi induced grayish phenotype in abdominal epidermis of *I. senegalensis*. (*A*) Expression levels of *broad* core region (sum of all zinc-finger isoforms). (*B*) Phylogenetic tree of *broad* zinc-finger domains based on their amino acid sequences. A neighbor-joining phylogeny is shown. Statistical support values for each clade are indicated in the order of bootstrap probability of the neighbor-joining analysis and bootstrap probability of the maximum-likelihood analysis from left to right, in which asterisks indicate values less than 50%. Deduced amino acid sequences of zinc-finger domains from other insects are modified from ref. 41. (*C*) RNAi phenotypes around the dorsal eighth abdominal segment. “All ZF isoforms” means using the equivalent mixture of dsRNA for seven *broad* zinc-finger isoforms. RNAi experiments were conducted at the early stage of the penultimate instar, and RNAi phenotypes were observed after ecdysis to the final instar. Numbers in parentheses indicate the number of individuals affected by RNAi/number of molted nymphs. White arrowheads indicate the grayish regions. (*D*) A magnified view of RNAi phenotype for *broad* BTB domain. Numbers beside the abdomens indicate the number of abdominal segments.

We searched for nymph-specific or adult-specific genes by edgeR analysis (false discovery rate [FDR] < 0.01), in which penultimate instar nymphs and adults were compared for the head, thorax, abdomen, and wings. Here, we used penultimate instar nymphs rather than final instar nymphs (Fig. 1*C*), considering that the gene-expression patterns may have changed significantly toward adult-like during the final nymphal instar (5). As a result, 64 nymph-specific contigs and eight adult-specific contigs that exhibited differential expression in all the body regions were identified (*SI Appendix*, Fig. S2). To further screen the candidate genes, we adopted the following three criteria: 1) for one group, minimum TPM (transcripts per million) value is no less than 2; 2) for the other group, maximum TPM value is less than 2; and 3) the minimum TPM value of one group is 1.5 times higher than the maximum TPM value of the other group. Using these criteria, eight nymph-specific contigs and seven adult-specific contigs were detected common to all four body regions (Fig. 1 *D* and *E* and Table 1). Using the Integrative Genomics Viewer (30), each contig sequence was carefully examined, and the full-length sequence was manually obtained (Table 1). Of the eight nymph-specific genes (referred to as *N1*-*N8*), *N4* and *N7* corresponded to the transcription factors *Kr-h1* and *broad*, respectively, and of the seven adult-specific genes (referred to as *A1*-*A7*), *A3* corresponded to the transcription factor *E93*. The annotation and the expression pattern of each gene are shown in Table 1, Figs. 2*A*, 3*A*, and 4*A*, and *SI Appendix*, Figs. S3 and S4. Three and eight isoforms were identified for *N4* (*Kr-h1*) and *N7* (*broad*), respectively, while only one isoform was identified for the remaining nymph-specific and adult-specific genes.

**Table 1.** Lists of nymph-specific and adult-specific contigs obtained by the screening criteria in this study

	Accession no.	Length (bp)	Description (species)	Blastx e-value
N1	LC634382	1,123	Uncharacterized secreted protein	
N2	LC634383	3,095	Peroxidase (*D. melanogaster*)	1 × 10^−86^
N3	LC634384	1,205	Uncharacterized protein	
N4	LC634385	6,962	**Transcription factor Krüppel homolog 1 (*D. melanogaster*)**	1 × 10^−109^
N5	LC634386	399	Uncharacterized secreted protein	
N6	LC634387	639	Uncharacterized secreted protein	
N7	LC634388–LC634395	1,641 to 7,876	**Transcription factor broad (*D. melanogaster*)**	8 × 10^−90^
N8	LC634396	896	CG4686 transmembrane protein (*D. melanogaster*)	2 × 10^−19^
A1	LC634397	1,769	Triglyceride lipase (*D. melanogaster*)	2 × 10^−56^
A2	LC634398	1,543	CG10407 takeout/JHBP family protein (*D. melanogaster*)	2 × 10^−44^
A3	LC634399	8,790	**Transcription factor E93 (*D. melanogaster*)**	6 × 10^−33^
A4	LC634400	1,915	Adenylate kinase 3 (*D. melanogaster*)	1 × 10^−48^
A5	LC634401	2,512	CG31344 ER-bound oxygenase (*D. melanogaster*)	4 × 10^−17^
A6	LC634402	1,525	Chitinase 10 (*D. melanogaster*)	2 × 10^−26^
A7	LC634403	1,850	CG9259 Ecdysteroid kinase-like protein (*D. melanogaster*)	4 × 10^−26^

Bold indicates the genes whose RNAi induced phenotypic effects on the abdominal epidermis of Odonata.

### Functional Analysis of Nymph-Specific and Adult-Specific Genes.

Next, we conducted electroporation-mediated RNAi of the nymph-specific and adult-specific candidate genes. For the nymph-specific genes, double-stranded RNA (dsRNA) was injected at the early stage of the penultimate nymphal instar, and the phenotype was observed after ecdysis to the final instar. For the adult-specific genes, dsRNA was injected at the early stage (stage 1) (31) of the final nymphal instar, and the phenotype was observed after adult emergence. In Odonata species, conventional RNAi does not work in the whole body, whereas in vivo electroporation after dsRNA/small interfering RNA injection can induce local gene suppression on the epidermal tissue around the region where the positive electrode was placed for electroporation (26, 27). Among the eight nymph-specific candidate genes, phenotypic effects were detected for two genes, *N4* (*Kr-h1*) and *N7* (*broad*) (*SI Appendix*, Fig. S5 and Table S2), whereas among seven adult-specific candidate genes, phenotypic effects were observed only for *A3* (*E93*) gene (*SI Appendix*, Fig. S6 and Table S2). Subsequently, we investigated the RNAi effects of *Kr-h1*, *broad*, and *E93* in detail.

### *Kr-h1* and JH Receptor *Met* Are Essential for Maintaining Nymphal Status in *I. senegalensis*.

*Kr-h1* of *I. senegalensis* had three isoforms (accession nos. LC634385, LC636406, and LC636407), one of which (*Kr-h1a*) was expressed until the penultimate nymphal instar and other isoforms were strongly expressed in the late embryonic stage (Fig. 2*A* and *SI Appendix*, Fig. S7) as reported in *Drosophila* (5, 32). RNAi of *Kr-h1* (targeting the region common to all isoforms) induced adult-like phenotypes on the nymphal cuticle (16/17 = 94%), of which especially remarkable was the light-blue markings on the eighth abdominal segment of males (white arrowhead; Fig. 2 *B* and *C*). Detailed observation on the surface fine structure using scanning electron microscopy (SEM) confirmed that RNAi of *Kr-h1* induced adult-like thinner bristles and small granular protrusions on the surface (*SI Appendix*, Fig. S8*A*), suggesting that *Kr-h1* is essential for maintaining nymphal status in *I. senegalensis*. When *Kr-h1* RNAi was performed in antepenultimate instar (nymphal instar just before the penultimate instar) nymphs, adult-like phenotypes were also induced (5/8 = 63%) (*SI Appendix*, Fig. S8 *B* and *C* and Table S2).

We also focused on the JH receptor gene to determine whether expression of *Kr-h1* in *I. senegalensis* is JH dependent as in other insects. In insects, the basic helix–loop–helix Per-ARNT-Sim (bHLH-PAS) protein gene *Met* is an intracellular receptor for JH (7). The expression of *Met* in *I. senegalensis* (accession no. LC634404) was detected at various developmental stages and body regions (Fig. 2*A*). RNAi of *Met* in penultimate instar nymphs induced adult-like phenotypes in final instar nymphs after ecdysis (13/14 = 93%), which closely resembled the RNAi phenotypes of *Kr-h1* (Fig. 2*C* and *SI Appendix*, Fig. S8*D*). The nymphs with phenotypes induced by RNAi of *Kr-h1* or *Met* failed in ecdysis around the RNAi region during adult emergence (*SI Appendix*, Fig. S8 *E* and *F*). It should be noted that RNAi of *Kr-h1* or *Met* in final instar nymphs did not cause any phenotypic effects in adults (*SI Appendix*, Fig. S8*G*).

In several insects, another bHLH-PAS protein called taiman (tai) forms a complex with Met to bind the JH response DNA motif and induces transcription of target genes (33–35). However, RNAi of *tai* in *I. senegalensis* (accession no. LC634405) in penultimate instar nymphs produced wound-like effects on final instar nymphs (10/10 = 100%), and the nymphs died soon after ecdysis (*SI Appendix*, Fig. S9), which were reminiscent of the lethal effects of *tai* RNAi in the red flour beetle *Tribolium castaneum* (36), the linden bug *Pyrrhocoris apterus* (37), and the cockroach *Blattella germanica* (38). In fruit flies and mosquitoes, *tai* is also important for ecdysteroid-signaling (5, 39), and *tai* is intimately involved in both JH and ecdysone pathways. The wound-like effects by *tai* RNAi may be due to an interference with molting in *tai* RNAi region, because ecdysteroid cannot act properly. RNAi of *tai* in *I. senegalensis* in final instar nymphs altered coloration of the adult light-blue regions and inhibited melanin pigmentation (6/10 = 60%) (*SI Appendix*, Fig. S9).

### *E93* Is Essential for Adult Morphogenesis in *I. senegalensis*.

Meanwhile, RNAi of *E93* in final instar nymphs changed the color of the adult abdominal epidermis to light brown (13/13 = 100%), which was reminiscent of the nymphal epidermis (Fig. 3). The surface fine structures of the *E93* RNAi individuals observed by SEM confirmed that in the *E93* RNAi regions (left side in Fig. 3*E*), bristles were thicker than in the control regions (right side in Fig. 3*E*), and small granular protrusions on the surface were inconspicuous, closely resembling the nymphal surface structure (Fig. 3 *C–G*). The nymph-like phenotypes induced by *E93* RNAi were also observed in the thorax of adults (*SI Appendix*, Fig. S10*A*). When *E93* RNAi was performed in penultimate instar nymphs, no phenotypic effect was observed in final instar nymphs (*SI Appendix*, Fig. S10*B*), whereas nymph-like epidermal phenotypes appeared in the subsequent emerged adults (*SI Appendix*, Fig. S10*C*). Previous studies showed that final instar nymphs of Odonata can be categorized into three developmental stages (stages 1, 2, and 3) based on the morphology of wing sheaths, and the middle stage of *I. senegalensis* can be further divided into three stages (stage 2a, 2b, and 2c) based on the morphology of compound eyes (31). The phenotypes of *E93* RNAi diminished when RNAi was conducted at stage 2 and after (*SI Appendix*, Fig. S10 *D* and *E*), presumably reflecting the increased expression level of *E93* at stage 2 and after in the abdomen (Fig. 3*A*).

### RNAi of *broad* Induces Unique Phenotypes in *I. senegalensis*.

As in other insects, the *broad* gene transcripts consisted of multiple isoforms with different 3′ end (*SI Appendix*, Fig. S11). The protein encoded by *broad* gene contains a core region consisting of a Broad-Complex-Tramtrack-Bric-à-brac (BTB) domain and a linker region that are commonly found among the isoforms and a C_2_H_2_ zinc-finger domain that is alternatively spliced. The C_2_H_2_ zinc-finger domains in the *broad* gene transcripts are classified into six clades (5, 40, 41). We identified seven zinc-finger domains from *broad* gene transcripts of *I. senegalensis* (accession nos. LC634388 through LC634395) (*SI Appendix*, Fig. S11), which were classified into five clades except for the cockroach-specific ZF6 clade (Fig. 4). When we also examined the published genomes of two Odonata species, *Ladona* (= *Libellula*) *fulva* (Anisoptera; Libellulidae; GenBank: GCA_000376725.2) (42) and *Ischnura elegans* (Zygoptera; Coenagrionidae) (43), seven zinc-finger domains were identified in both species (Fig. 4*B* and *SI Appendix*, Fig. S11*A*). Therefore, we named seven zinc-finger domains conserved in Odonata as ZFa-ZFg in order from the 5′ side (*SI Appendix*, Fig. S11*A*). In *I. senegalensis*, the expression patterns of the isoform were similar to each other, among which *ZFd* and *ZFe* exhibited relatively high expression levels (*SI Appendix*, Fig. S11*B*). All isoforms were outstandingly expressed in caudal gills, a nymph-specific organ (Fig. 1*C*, arrowheads), during the final nymphal instar (Fig. 4*A* and *SI Appendix*, Fig. S11*B*). Since the caudal gills are spontaneously excised by stimulation like electroporation, the effect of RNAi could not be examined.

RNAi targeting the *broad* common region (BTB domain or linker region) induced a grayish pigmentation in the nymphal epidermis of both males and females (white arrowheads, Fig. 4 *C* and *D*), which was distinct from the phenotypes of *Kr-h1* RNAi. The surface fine structures exhibited no obvious differences between the *broad* RNAi regions and the control regions (*SI Appendix*, Fig. S12 *A–C*). Like *Kr-h1* RNAi, the nymphs with phenotypes induced by *broad* RNAi failed in ecdysis around the RNAi region upon adult emergence (*SI Appendix*, Fig. S12*D*). It should be noted that *broad* RNAi in final instar nymphs caused no phenotypic effects in adults (*SI Appendix*, Fig. S12*E*). Single RNAi experiments specifically targeting either *ZFd*, *ZFe*, or *ZFf* exon were performed to identify the function of each isoform, but none of them affected the epidermal pigmentation. On the other hand, double RNAi of *ZFd* and *ZFe* and multiple RNAi of seven isoforms induced the grayish epidermal phenotype as observed in RNAi targeting the common region (Fig. 4*C*), suggesting that these isoforms have redundant function in the epidermal pigmentation.

Because *broad* is known to be involved in wing development in Hemiptera (18, 44), Orthoptera (14), and Blattodea (19), we attempted to perform RNAi experiments against wings of Odonata. However, when electroporation was applied at 25 V, the same voltage as that applied to the epidermis, wing formation was inhibited even in the negative control (*SI Appendix*, Fig. S13*A*). When the voltage was lowered to 10 V, the detrimental effects in the negative control were attenuated, but the phenotypic effects of *Kr-h1* RNAi and *broad* RNAi were not observed (*SI Appendix*, Fig. S13*B*). These results suggested that the electroporation-mediated RNAi is not applicable to the wings of penultimate instar nymphs of *I. senegalensis*.

### Gene-Expression Profiles Associated with RNAi of *Kr-h1*, *broad*, and *E93*.

**Fig. 5.**
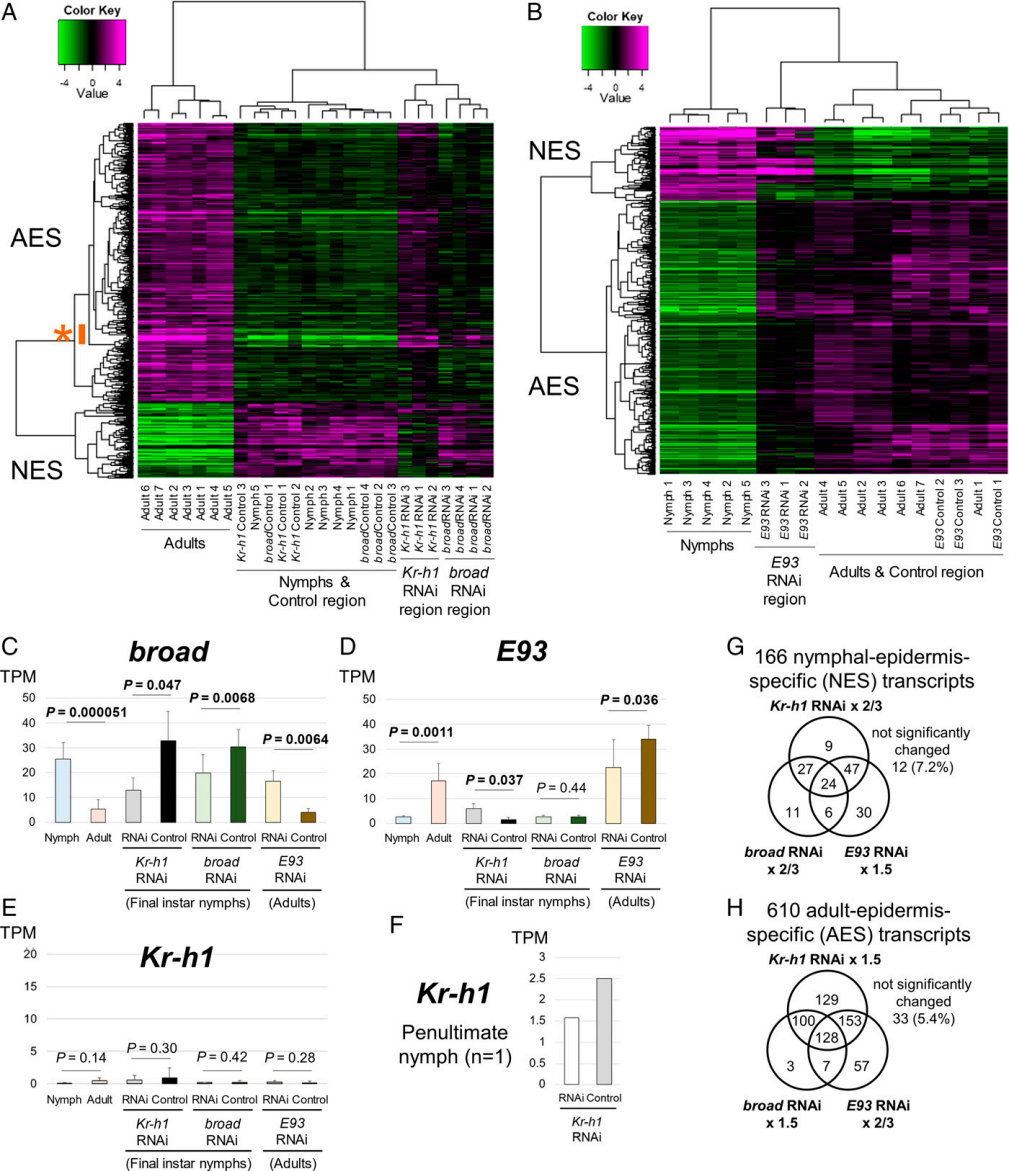
Summary of RNA-sequencing in *Kr-h1* (*n* = 3), *broad* (*n* = 4), and *E93* (*n* = 3) RNAi individuals. (*A* and *B*) Clustering dendrogram and heatmap on the differential gene expression of 166 NES and 610 AES transcripts. Magenta color is in high expression level, while green color is in low expression level. Color key value indicates the log_2_ fold change in the expression levels. Asterisk indicates genes whose expressions were induced by both *Kr-h1* RNAi and *broad* RNAi individuals. (*A*) *Kr-h1* *(n* = 3) and *broad* (*n* = 4) RNAi individuals. (*B*) *E93* (*n* = 3) RNAi individuals. (*C*–*E*) Expression level changes of *broad* (sum of all isoforms) (*C*), *E93* (*D*), and *Kr-h1* (*E*) genes after RNAi treatments. (*F*) Expression level changes of *Kr-h1* between *Kr-h1* RNAi region and control region in the penultimate instar. (*G* and *H*) Venn diagrams of 166 NES (*G*) and 610 AES (*H*) transcripts. Each circle indicates genes satisfying these criteria: 1) the average expression in the RNAi regions exhibits a more-than-1.5-fold change compared with the average expression in the control regions, and 2) all individuals showed the same tendency for increase or decrease. *P* value between untreated nymphs and adults was calculated by Student’s *t* test, while *P* value between RNAi regions and control regions was calculated by paired *t* test. Error bars are SD.

In *I. senegalensis*, as in other insects, it was confirmed that *Kr-h1* RNAi induced precocious adult phenotypes and *E93* RNAi retained nymphal phenotypes. Meanwhile, the morphological phenotypes of *broad* RNAi were difficult to interpret. Therefore, gene-expression patterns of the abdominal epidermis caused by RNAi of *Kr-h1* (*n* = 3), *broad* (*n* = 4), and *E93* (*n* = 3) were analyzed by RNA-sequencing. For comparison, samples of the abdominal epidermis from untreated final instar nymphs (1 d after nymphal ecdysis) and adults (1 to 2 d after adult emergence) were also used. We identified 166 nymphal-epidermis-specific (NES) transcripts (158 genes plus eight isoforms) and 610 adult-epidermis-specific (AES) transcripts (595 genes plus 15 isoforms) from the untreated final instar nymphs (*n* = 12) and adults (*n* = 10) based on the following criteria: 1) edgeR analysis (FDR < 0.01), and 2) the minimum TPM value of one group is more than 1.5 times higher than the maximum TPM value of the other group (Dataset S1). By focusing on the NES and AES genes, we constructed hierarchical cluster dendrograms, which confirmed that the gene-expression patterns of the *Kr-h1* RNAi regions became close to those of untreated adults (Fig. 5*A*). Notably, some of the AES genes were strongly up-regulated in the *broad* RNAi regions, uncovering that the gene-expression patterns of the *broad* RNAi regions were partially similar to those in adults (Fig. 5*A*, asterisk). By contrast, the gene-expression patterns of the *E93* RNAi regions were globally similar to those of final instar nymphs (Fig. 5*B*).

### Screening of Candidate Downstream Genes for *Kr-h1*, *broad*, and *E93*.

We confirmed that *broad* RNAi certainly reduced the expression levels of *broad* and so did *E93* RNAi the expression levels of *E93* (Fig. 5 *C* and *D* and *SI Appendix*, Fig. S14 *A* and *B*). As reported in lepidopteran species (45), since the RNAi regions exhibit mosaicism, the samples from the RNAi regions contain both affected and unaffected cells, so the reduction of the expression levels cannot be high inherently. On the grounds that the RNAi region and the control region were compared within the same individual, we tested the statistical significance of the difference by paired *t* test. We examined how these three transcription factors interact with each other. RNAi of *Kr-h1* reduced the expression of *broad* and increased the expression of *E93* (Fig. 5 *C* and *D* and *SI Appendix*, Fig. S14 *A* and *B*), as reported in other hemimetabolous insects (5). RNAi of *broad* affected neither the expression of *Kr-h1* nor the expression of *E93* (Fig. 5 *D* and *E* and *SI Appendix*, Fig. S14 *B* and *C*). RNAi of *E93* increased the expression of *broad* but did not affect the expression of *Kr-h1* (Fig. 5 *C* and *E* and *SI Appendix*, Fig. S14 *A* and *C*). While the expression levels of *Kr-h1* were consistently very low in final instar nymphs, the expression levels of *Kr-h1* in penultimate instar nymphs were substantial and reduced by *Kr-h1* RNAi (Fig. 5 *E* and *F* and *SI Appendix*, Fig. S14*C*).

Next, we surveyed target genes for the three transcription factors *Kr-h1*, *broad*, and *E93* (Dataset S1). More than 90% of both the NES and AES genes were consistently affected in all individuals by RNAi of at least one of the three transcription factors, while exhibiting a more-than-1.5-fold change in the average expression level in the RNAi regions compared with the control regions (Fig. 5 *G* and *H*). For example, the expression of 24 NES genes (Fig. 5*G*; as an example, NES013: *Cuticular protein RR1-type CPR-L5* is shown in *SI Appendix*, Fig. S14*D*) increased more than 1.5-fold by *E93* RNAi but decreased to less than 2/3 by *Kr-h1* and *broad* RNAi, whereas the expression of 128 AES transcripts (125 genes plus three isoforms) (Fig. 5*H*; as an example, AES021: *Cuticular protein CPCFC-A2* is shown in *SI Appendix*, Fig. S14*E*) increased more than 1.5-fold by the RNAi of *Kr-h1* and *broad* but decreased to less than 2/3 by the RNAi of *E93*. Since the expression of *Kr-h1* and *E93* is not affected by *broad* RNAi, these results indicate that *broad* regulates a substantial number of NES (24 + 27 + 6 + 11 transcripts; 68/166) and AES (128 + 100 + 7 + 3 transcripts; 238/610) genes without being mediated by *Kr-h1* or *E93*. In other words, a number of NES genes (9 + 47 + 30 transcripts; 86/166) (Fig. 5*G*; as an example, NES038: *Cuticular protein CPLCP-L1* is shown in *SI Appendix*, Fig. S14*F*) and AES genes (129 + 153 + 57 transcripts; 339/610) (Fig. 5*H*; as an example, AES050: *1-Acylglycerol-3-phosphate O-acyltransferase 2* is shown in *SI Appendix*, Fig. S14*G*) were affected by RNAi of *E93* and/or *Kr-h1* but not by RNAi of *broad*, suggesting that *E93* and *Kr-h1* regulate the expression of several genes without involvement of *broad*. It should be noted that some NES genes (9 + 27 transcripts) and AES genes (129 + 100 transcripts) were affected by RNAi of *Kr-h1* but not by RNAi of *E93* in our criteria (Fig. 5 *G* and *H*).While some of these genes are likely to be near the border of the criteria, at least some of them showed marked effects by RNAi of *Kr-h1* but little or no effects by RNAi of *E93* (as an example, NES038: *Cuticular protein CPLCP-L1* is shown in *SI Appendix*, Fig. S14*F* and Dataset S1), suggesting that *Kr-h1* may partly regulate the nymphal status independently of *E93*. Although 11 NES transcripts (NES055a, NES055b, NES058, NES095, NES097, NES098, NES115, NES119, NES121, NES143, and NES152) and three AES genes (AES135, AES162, and AES572) were only affected by RNAi of *broad* in our criteria (Fig. 5 *G* and *H*), similar expression change was observed by RNAi of *Kr-h1* in all these genes (but they did not meet the criteria) (Dataset S1). Since *broad* is induced by *Kr-h1* (Fig. 5*C*), these genes are likely to be controlled by both *Kr-h1* and *broad*.

Gene ontology analyses revealed that NES genes contained many structural molecule proteins (e.g., cuticular proteins), whereas AES genes contained many enzymes, binding proteins, transporters, and structural molecule proteins (*SI Appendix*, Fig. S15*A*). We confirmed that a variety of genes were affected by RNAi of *broad* (*SI Appendix*, Fig. S15*A* and Dataset S1). Among NES and AES genes, we identified 47 and 26 cuticular proteins, respectively (*SI Appendix*, Fig. S15*B* and Dataset S1). Based on the characteristic motif classification (46), cuticular protein genes with RR1 motif were predominantly found in NES genes, while cuticular protein genes with CPCFC motif were identified only from AES genes (*SI Appendix*, Fig. S15*B*). Interestingly, most of AES cuticular protein genes were down-regulated by *broad* (*SI Appendix*, Fig. S15*B* and Dataset S1). Nymphal and adult-specific cuticular genes seem to be involved in the formation of their characteristic surface structures (Fig. 3 *C–G* and *SI Appendix*, Fig. S8 *A* and *D*). Moreover, 16 genes involved in pigmentation (four genes for melanin, one gene for ommochrome, three genes for ommochrome, pteridine, and uric acid, and eight genes for pteridine and uric acid) were identified from AES genes (*SI Appendix*, Fig. S15*C* and Dataset S1). Because light-blue coloration in adult males is produced by coherent light-scattering due to the light-scattering granules containing pteridine pigments and the light-absorbing granules containing ommochrome pigments and black coloration in adults is produced by melanin pigments (47, 48), these genes may be involved in the formation of colorful adult pigmentation. Furthermore, among NES or AES genes, 12 transcription factors (e.g., *Blimp-1* and *HR4*) and 19 signaling receptor-related proteins (e.g., *hedgehog* and *EGFR*) were identified (*SI Appendix*, Fig. S15 *D* and *E* and Dataset S1). The biological role of these genes in metamorphosis deserves further study.

### Gene-Expression and Functional Analysis in Pied Skimmer Dragonfly *Pseudothemis zonata*.

**Fig. 6.**
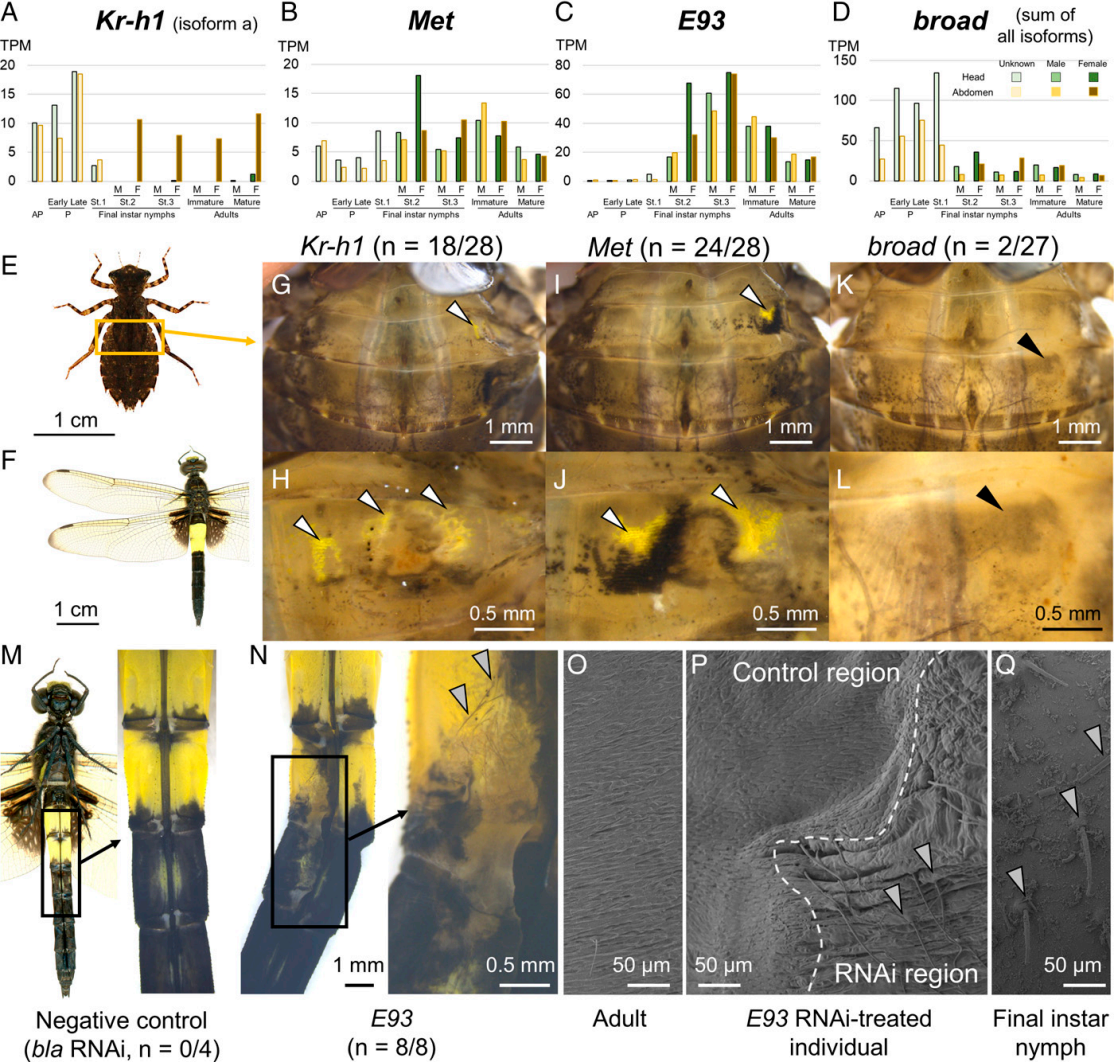
Molecular mechanisms underlying metamorphosis in the pied skimmer dragonfly *P. zonata*. (*A*–*D*) Expression levels. (*A*) *Kr-h1*, (*B*) *Met*, (*C*) *E93*, and (*D*) *broad*. M, F, AP, and P indicate male, female, antepenultimate instar nymphs, and penultimate instar nymphs, respectively. (*E*) A final instar nymph. (*F*) Adult male with yellow coloration on the third and fourth abdominal segments. (*G*–*L*) RNAi phenotypes in the final instar nymphs. (*G* and *H*) *Kr-h1*, (*I* and *J*) *Met*, and (*K* and *L*) *broad*. (*H*, *J*, and *L*) show magnified views of *G*, *I*, and *K*, respectively. White and black arrowheads indicate adult-specific yellow coloration and grayish pigmentation, respectively. (*M*) Ventral RNAi phenotypes of negative control (*bla*). (*N*) Ventral RNAi phenotypes of *E93*. (*O*–*Q*) SEM observation on the abdominal surface of *E93* RNAi individuals. (*O*) An adult without any treatments. (*P*) *E93* RNAi individual. *E93* RNAi region is on the right side of the photo. (*Q*) A final instar nymph without any treatments. Gray arrowheads indicate nymph-specific bristles on the abdominal surface.

The four genes, *Kr-h1*, *Met, E93*, and *broad*, which were shown to affect metamorphosis in the blue-tailed damselfly *I. senegalensis*, were also analyzed in a phylogenetically distant dragonfly *Pseudothemis zonata* (Anisoptera; Libellulidae) (accession nos. LC634406–LC634416). As in *I. senegalensis*, eight isoforms of *broad* (Fig. 4*B* and *SI Appendix*, Fig. S16) (accession nos. LC634409–LC634416) and two isoforms of *Kr-h1* were identified in *P. zonata* (*SI Appendix*, Fig. S7) (accession nos. LC634406 and LC636408). The expression patterns of these genes in *P. zonata* were similar to those in *I. senegalensis*, except that *Kr-h1* was highly expressed in the abdomen of females even after the final nymphal instar (Fig. 6 *A–D* and *SI Appendix*, Fig. S7). *Kr-h1* expression in female final instar nymphs could be related to JH-dependent reproductive activities, such as the production of yolk proteins (5).

Adult-specific yellow markings were induced by the RNAi of *Kr-h1* (28/41 = 68%) or *Met* (29/33 = 88%) when RNAi was conducted in the penultimate or antepenultimate instar nymphs (Fig. 6 *E–J* and *SI Appendix*, Fig. S17 and Table S2). The effect of *broad* RNAi was subtle, only slightly increasing blackness in several individuals (4/30 = 13%) (Fig. 6 *K* and *L* and *SI Appendix*, Fig. S17 and Table S2). By contrast, *E93* RNAi in final instar nymphs produced nymph‐like coloration and surface structures (Fig. 6 *M–Q*; nymph-specific bristles are indicated by gray arrowheads) in adults (8/8 = 100%), as observed in *I. senegalensis*.

## Discussion

### The Functional Role of the MEKRE93 Pathway in Metamorphosis of Odonata.

Comprehensive gene-expression analysis and RNAi functional analysis confirmed that three transcription factors, *Kr-h1*, *broad*, and *E93*, are essential for metamorphosis in Odonata, at least in the abdominal epidermis. Although dragonflies and damselflies greatly change their morphology from nymphs (often called larvae) to adults, the presence of *broad* expression from embryogenesis until the final nymphal instar is consistent with the cases of other hemimetabolous insects. Among hemimetabolous insects, such a large-scale transcriptomic analysis during metamorphosis has been reported only for cockroaches (49), and the comprehensive screening of metamorphosis-related genes with gene functional analysis as reported in this study is unique.

In some insect species such as the fruit fly *D. melanogaster*, adult epidermal cells proliferate from the histoblast nests, while in other insect species such as the tobacco hornworm *Manduca sexta*, the larval epidermal cells persist through metamorphosis and synthesize larval, pupal, and adult cuticles continuously (5, 50). In Odonata, the RNAi phenotype by electroporation shows finely mosaic patterns and varies between individuals, suggesting that the development of adult epidermal cells is likely to be derived from larval epidermal cells.

**Fig. 7.**
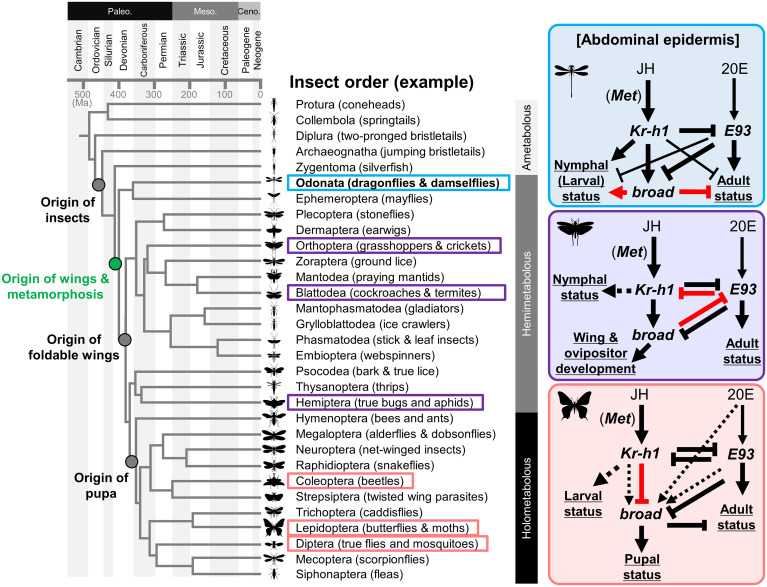
Summary of changes in the interactions of three transcription factors controlling insect metamorphosis. Insect phylogeny is based on refs. 21 and 63. The blue, purple, and red boxes represent Odonata (this study), other hemimetabolous insects (reviewed in ref. 5), and holometabolous insects (reviewed in ref. 5), for which the interactions between the three transcription factors have been examined. The red arrows and lines indicate the unique relationships between the genes in each group. In *Drosophila*, exogenous JH up-regulates *broad* through *Kr-h1* during pupa-to-adult metamorphosis (64). It should be noted that the interactions of three transcription factors of Odonata have been confirmed only in the abdominal epidermis.

In other insects, *Met* and *Kr-h1* RNAi were reported to cause precocious metamorphosis (7–9, 44, 51–53), and *E93* RNAi showed retention of larval/nymphal morphology and larva-/nymph-specific gene-expression pattern (12, 14). The rewiring of the relationship of genes involved in metamorphosis during insect evolution is shown in Fig. 7. Comparison of gene-expression patterns in RNAi individuals showed that *Kr-h1* suppresses *E93* (Figs. 5 and 7 and *SI Appendix*, Fig. S18), as reported in other insects (12–16), indicating that the central part of the MEKRE93 pathway is conserved in Odonata. In the mayfly *Cloeon viridulum*, both *Kr-h1* and *broad* were highly expressed at the nymphal stage (54). Although functional analysis of these genes and expression of *E93* have not been reported from Ephemeroptera, these results are concordant with our results in Odonata.

Unlike other insect groups (5, 12, 14), suppression of *Kr-h1* expression by *E93* was not observed in this study: the expression of *Kr-h1* did not increase in the RNAi regions of *E93*, at least in the abdominal epidermis during the final nymphal instar (Figs. 5 and 7 and *SI Appendix*, Fig. S14), despite the retainment of nymphal morphology (Figs. 3 and 6). This result can be interpreted in two ways. First, dragonflies and damselflies are different from other insect groups. Alternatively, because previous studies used systemic RNAi, the suppression of the gene expression in one tissue can be affected by other tissues indirectly. For example, an increase in *E93* in the corpora allata (JH-producing organ) at the onset of metamorphosis may be involved in the cessation of JH synthesis, and systemic *E93* RNAi could affect the corpora allata, allowing production of JH, thereby increasing *Kr-h1* expression. In each body part (head, thorax, abdomen, and wings), the expression of *E93* was almost undetectable in the penultimate nymphal instar, detected at early stage 1 of the final nymphal instar, and drastically up-regulated at stage 2 of the final nymphal instar and on (Figs. 3*A* and 6*C*). The effect of *E93* RNAi was confirmed up to early stage 2 of the final nymphal instar (*SI Appendix*, Fig. S10*E*), which was more than 2 wk after the depletion of *Kr-h1* expression (Fig. 2*A*), suggesting that the loss of *Kr-h1* expression is not attributable to *E93* expression.

### Function of *broad* Gene in Odonata and Evolution of Metamorphosis in Insects.

The most-noteworthy finding in this study is the role of *broad* gene in Odonata. In holometabolous insects, *broad* is specifically expressed in the pupal development and is essential for pupation (55–58). Meanwhile, in hemimetabolous insects without pupal stage, *broad* gene is highly expressed during the nymphal stage and is mainly involved in the development of adult wings and ovipositor (5, 12, 14, 18, 44). It should be noted that *broad* gene has also been identified in ametabolous firebrat *Thermobia domestica* (Zygentoma), although its function has not been examined (59). In Odonata, *broad* was exclusively expressed during the nymphal period, and RNAi of *broad* induced adult-like pigmentation (Figs. 4 *C* and *D* and 6 *K* and *L*) but did not significantly change the nymphal surface structures (*SI Appendix*, Fig. S12). It should be noted that some change in pigmentation patterns by RNAi of *broad* has also been reported from the milkweed bug (18). In the *broad* RNAi nymphs, expression levels of both *Kr-h1* and *E93* were unchanged, while expression levels of many of NES and AES genes were affected (Fig. 5). These results strongly suggest that *broad* gene regulates these genes without being mediated by *Kr-h1* and *E93* (Fig. 7 and *SI Appendix*, Fig. S18). The contribution of *broad* to the repression of genes involved in adult status is similar to that in holometabolous insects (5), while the contribution of *broad* to the induction of genes involved in nymphal status is, to our knowledge, previously undescribed (Figs. 5 and 7 and *SI Appendix*, Fig. S18). Our results suggest that *broad* is a major player downstream of *Kr-h1* in maintaining juvenile traits and suppressing adult traits in nymphs of Odonata. In adults of Odonata, *E93* represses the *broad* gene as in other insects (Figs. 5 and 7 and *SI Appendix*, Figs. S14*A* and S18) (5).

The RNAi phenotypes of *broad* were different from those of *Kr-h1* (Figs. 2 and 4 and *SI Appendix*, Figs. S8 and S11), which can be explained as follows. Because RNAi of *broad* does not induce *E93* expression (Fig. 5 and *SI Appendix*, Fig. S14*B*), the adult status controlled by *E93* cannot be reproduced (Fig. 7). In addition, since *broad* regulates a subset of nymph-specific and adult-specific genes without being mediated by *E93* or *Kr-h1* (Figs. 5 and 7), RNAi of *broad* may end up with unique phenotypes that are distinct from both nymphs and adults (Fig. 4).

It has been argued that functional differences in *broad* gene across diverse insect groups are important in understanding the evolution of metamorphosis (5, 60, 61). Previously, *broad* gene was shown to be mostly involved in the development of wings and ovipositor in hemimetabolous insects based on the RNAi phenotypes, but its role in other tissues has been unknown. In this study, we demonstrate that in Odonata species whose body coloration differs between adults and nymphs, the *broad* gene is involved in the development of the abdominal epidermis, at least to some extent. We also suggest that plausibly, the effects of *broad* RNAi may be different across insect groups, on the grounds that *broad* suppresses *E93* in other hemimetabolous insects (Fig. 7). In holometabolous insects, another major change must have occurred: *Kr-h1* does not induce but suppresses *broad* (5). Modification and rewiring of the relationship across the transcription factors *Kr-h1*, *broad*, and *E93* may have contributed to the evolution of insect metamorphosis, and therefore, it is necessary to clarify when and how these changes have occurred in the evolution of diverse insects. In this study, we also identified an array of genes potentially regulated by *broad* gene in Odonata (Fig. 5 *G* and *H* and *SI Appendix*, Figs. S14 *D–G* and S15 and Dataset S1). Investigation of expression, regulation, and functions of these genes in comparison with other insect systems would provide insight into the evolutionary origin of insect pupa and holometaboly.

## Materials and Methods

Complete details on the materials and methods are available in *SI Appendix*.

Nymphs and adults of *I. senegalensis* and *P. zonata* used in this study were collected in Tsukuba, Ibaraki, Japan. Some eggs of *I. senegalensis* were obtained from wild mature females and reared in the laboratory as described (25). Total RNA was extracted from the freshly prepared samples using RNAiso Plus (Takara Bio), and complementary DNA libraries were constructed using TruSeq RNA Sample Preparation Kits version 2 (Illumina) and sequenced by HiSeq (Illumina). The sample information and RNA-sequencing reads are shown in *SI Appendix*, Table S1. Transcriptome analyses were performed as described (62). Electroporation-mediated RNAi was conducted as described previously (26, 27). As a negative control, we used a dsRNA targeting *β-lactamase* (*bla*) gene. The primer sets to produce templates for dsRNA synthesis are shown in *SI Appendix*, Table S3. RNAi targeting the multiple genes was performed using an equivalent mixture of 100 μM dsRNA solutions (i.e., final concentration was 50 μM each for the RNAi of two genes). The overall results of RNAi experiments were summarized in *SI Appendix*, Table S2.

## Supplementary Material

Supplementary File

Supplementary File

## Data Availability

DNA sequences and raw Fastq data have been deposited in DNA Data Bank Japan Read Archive (LC634382–LC634416, LC636406–LC636408, DRR278508–DRR278641, and DRR300699–DRR300702). All other study data are included in the article and/or supporting information.
