# Supplementary material for: Molecular mechanisms underlying metamorphosis in the most-ancestral winged insect
Source: Proc Natl Acad Sci U S A. 2022 Feb 25;119(9):e2114773119. doi: 10.1073/pnas.2114773119 (PMC8892354; doi:10.1073/pnas.2114773119)
Supplement: Supplementary File [file pnas.2114773119.sapp.pdf]

PNAS
www.pnas.org

**Supplementary Information for**

# Molecular mechanisms underlying metamorphosis in the most ancestral winged insect

Genta Okude, Minoru Moriyama, Ryouka Kawahara-Miki, Shunsuke Yajima, Takema Fukatsu, Ryo Futahashi

Genta Okude
Email: gentaokude@gmail.com

Ryo Futahashi
Email: ryo-futahashi@aist.go.jp

**This PDF file includes:**

SI Materials and Methods
Figures S1 to S18
Tables S1 to S3
Legends for Dataset S1
SI References

**Other supplementary materials for this manuscript include the following:**

Dataset S1

## SI Materials and Methods

### Insect collection and rearing

Here we adopt the term "nymph" instead of "larva" to distinguish between holometabolous and hemimetabolous insects, though the majority of previous studies on Odonata preferred to the term "larva" because dragonflies and damselflies drastically change the external morphology through metamorphosis unlike most other hemimetabolous insects (1, 2). It should be noted that we used the term "antepenultimate instar", "penultimate instar", and "final instar", instead of the precise ordinal number of instars, because the number of nymphal instars is generally not fixed in Odonata (3).

Nymphs and adults of *Ischnura senegalensis* and *Pseudothemis zonata* used in this study were collected in Tsukuba, Ibaraki, Japan. For *I. senegalensis*, nymphs were also reared from eggs obtained from adult females. The nymphs of *I. senegalensis* were individually kept in 48-well, 24-well, or 12-well plates with water depending on the nymphal size and were fed with *Artemia* brine shrimp every day as described previously (3,4). The nymphs of *P. zonata* were kept in a group because they rarely cannibalize and fed with bloodworms and/or *Tubifex* worms at least twice a week (4).

The last three nymphal instars (antepenultimate, penultimate, and final instar) of *I. senegalensis* and *P. zonata* were determined by the size of wing sheaths (2, 3, 5). The final nymphal instar of Odonata can be categorizing into three stages (stage 1, 2, and 3) based on the morphology of wing sheaths, and in *I. senegalensis*, the middle stage can be further divided into three stages (stage 2a, 2b, and 2c) according to the morphology of compound eyes (3, 6). Nymphal sexing of *I. senegalensis* was performed on the basis of sex-specific morphological traits observed on the ventral side of the abdominal tip (3). *I. senegalensis* adult females have two color morphs, the male-mimicking color morph called androchrome and the female-specific color morph called gynochrome, but the color morphs of female cannot be determined during nymphal stage. Male of *P. zonata* was identified after stage 2 of final instar nymphs based on the presence of accessory genitalia in the ventral side of the nymphal abdomen (5).

### Transcriptome analysis

To construct a comprehensive catalog of expressed genes, RNA-sequencing was conducted for various developmental stages, body regions, and sexes. In *I. senegalensis*, multiple individuals were subjected to each sample preparation for eggs, pronymphs, and 1$^{st}$ instar nymphs, whereas a whole individual was used for each sample preparation of 4$^{th}$-7$^{th}$ instar nymphs (*SI Appendix,* Fig S1, Table S1). It should be noted that the final nymphal instar is generally between 10 and 12 in *I. senegalensis* (3). The penultimate and the final instar nymphs were dissected into head, thorax, abdomen, wing buds, and caudal gills for extracting total RNA samples. we selected six different time points during the final nymphal instar, among which Day 3, 11, 15, 19, 23, and 25 were

corresponded to stage 1 (early), 1 (late), 2a, 2b, 2c, and 3, respectively, judging from the morphological characteristics. We used only males for samples of penultimate, and final instar nymphs. Immature (Day 1) and mature adults (filed collected) were dissected into head, thorax, abdomen, and wings for extracting total RNA samples. *I. senegalensis* females have two color morphs, the male-mimicking color morph called androchrome and the female-specific color morph called gynochrome, and we used both (*SI Appendix,* Fig S1, Table S1).

In *P. zonata*, head and abdomen of one individual were used for each sample of antepenultimate instar nymphs, penultimate instar nymphs, final instar nymphs, immature adults (Day 1), and mature adults (field collected). Because we could not determine the sex by the first stage of the final nymphal instar, we distinguished the sex of samples after entering the middle stage of the final nymphal instar (*SI Appendix,* Table S1).

To examine the effect of RNAi on gene expression in *I. senegalensis*, the epidermis of RNAi region with clear phenotypic effect (around the left 8$^{th}$ abdominal segment) and the epidermis of control region without RNAi effect (around 4$^{th}$ and 5$^{th}$ abdominal segments) were dissected by micro scissors 1-3 days after nymphal ecdysis or adult emergence (*SI Appendix,* Table S1). The 4$^{th}$ and 5$^{th}$ abdominal epidermis of nymph or adult without RNAi treatment was also used as a control. Fat bodies and tracheas attached to the internal side of the dissected epidermis were removed as much as possible.

Total RNA was extracted from the freshly prepared samples using RNAiso Plus (Takara Bio) following the manufacturer's protocol. Using 0.1-1 μg of total RNA per sample as template, cDNA libraries were constructed using TruSeq RNA Sample Preparation Kits v2 (Illumina) and sequenced by HiSeq (Illumina). The sample information and the number of RNA-sequencing reads are shown in *SI Appendix,* Table S1.

The quality of obtained data was checked using the FastQC program (7). Adaptor and low-quality sequences were trimmed using the Trimmomatic program (8). Subsequently, the trimmed reads were subjected to *de novo* assembly using the Trinity program v. 2.4.0 (9). After automatic assembling, we checked and manually corrected the sequences of the focused genes (*Kr-h1*, *broad*, *E93*, *Met*, *tai*, 8 nymph-specific genes, 7 adult-specific genes, 158 nymphal-epidermis-specific (NES) genes, and 595 adult-epidermis-specific (AES) genes) using the Integrative Genomics Viewer (10). After revising the sequence, mapping was performed using the Salmon program (version 1.5.1) (11), whereby transcript expression levels were estimated as TPM (transcripts per million) values. Each of the NES and AES gene was assigned a serial identification number in the order of FDR value by edgeR program (12) in R software (version 3.5.1).

For annotation of the reference contigs, each contig sequence was subjected to BLASTx search ($P < 1e^{-5}$) (13) against *Drosophila* protein database (Flybase ver. 6.42) and the Swiss-Prot database (release 2021_04). SignalP 5.0 (14) was used for signal peptide prediction. Heatmaps on the differential gene expression were described by using the Heatmap.3 program in R software

(version 3.5.1) (15). Gene ontology (GO) terms was assigned using FlyBase GO classification. Classification of cuticular protein genes was based on having a signal peptide sequence and a characteristic protein motif (16), and all cuticular protein genes were assigned as structural molecule activity (GO:0005198) in GO term.

**Identification of differentially expressed genes**

Differentially expressed genes were extracted using the edgeR program (12) in R software (version 3.5.1) (15). We also set the following three criteria to further screen for highly and exclusively expressed nymph-specific genes in each body region (head, thorax, abdomen, and wing/wing buds): (i) TPM values for all three samples from penultimate instar nymphs were larger than 2, (ii) TPM values for all six samples from adults were smaller than 2, and (iii) The minimum TPM value of penultimate instar nymphs is more than 1.5 times the maximum TPM value of adults. Adult-specific genes were extracted vice versa.

To examine the genes affected by RNAi treatment, differentially expressed genes were extracted using the dependent *t*-test for paired samples from RNAi and control regions from the same individual. The following two criteria were used to further screen for genes affected by RNAi: (i) the average expression in the RNAi regions exhibits a more than 1.5-fold change compared with the average expression in the control regions, and (ii) all individuals showed the same tendency for increase or decrease.

**RNAi experiments**

Electroporation-mediated RNAi was conducted as described previously (4). The primer sets to produce templates for dsRNA synthesis were designed using the primer3 program version 4.1.0 (17) (*SI Appendix,* Table S3). Total RNA was extracted from the freshly prepared samples using RNAiso Plus (Takara Bio) following the manufacturer's protocol. Using 1 μg of total RNA per sample as template, cDNA was synthesized using SuperScript II Reverse Transcriptase (Thermo Fisher Scientific). The target sequences were amplified using the synthesized cDNA and the designed primer set and cloned into the pT7Blue T-vector (Novagen) using the DNA Ligation Kit Ver. 2.1 (Takara Bio). The plasmid was transformed into *E. coli* competent cells and a single colony after overnight incubation was picked up. The insert region was PCR-amplified using primers on the vector (Uni19: 5′-GTT TTC CCA GTC ACG ACG T-3′ and Rev20: 5′-AGC TAT GAC CAT GAT TAC GC-3′). After the cloned sequence was confirmed by Sanger sequencing using BigDye Terminator v3.1 Cycle Sequencing Kit (Applied Biosystems) and 3130xl Genetic Analyzer (Applied Biosystems), the insert was PCR-amplified using vector primers containing the T7 polymerase promoter sequence (T7-F: 5' - TAA TAC GAC TCA CTA TAG GGA GAC TAG TCA TAT GGA T - 3' and T7-R: 5'- TAA TAC GAC TCA CTA TAG GGA GAC CCG GGG ATC CGA T - 3') (18, 19).

The PCR product was purified using the QIAquick PCR purification Kit (QIAGEN), and eluted with 50 µL of distilled water. The eluted DNA solution was concentrated to approximately 10 µL using a centrifugal evaporator. A total of 1000 ng template DNA was used, and dsRNA was synthesized by *in vitro* transcription using MEGAscript RNAi kit (Thermo Fisher Scientific). Synthesized dsRNA was eluted with 100 µL elution buffer, and diluted to 1000 ng/µL with elution buffer. The quality of dsRNA was confirmed by electrophoresis on a 1.5% agarose gel, and dsRNA was stored at -20 °C until use.

A glass capillary (calibrated micropipette 1-5 µL; Drummond) was pulled by using a glass needle puller (PN-3, NARISHIGE). The tip of the pulled capillary was placed onto a double-sided adhesive tape and was broken with forceps. The capillary was set to an injector (IM-12, NARISHIGE) and dsRNA solution was loaded to the prepared capillary. As for *I. senegalensis*, a nymph was anesthetized covered with a wet paper on crushed ice for 50-70 seconds. 1 µL of dsRNA solution was injected into the inter-segmental membrane between the $7^{th}$ and $8^{th}$ abdominal segment for RNAi in the abdomen or between the prothorax and synthorax (fused mesothorax and metathorax) for RNAi in the thorax. As for *P. zonata*, a small hole was made with a fine needle in the inter-segmental membrane between the $4^{th}$ and $5^{th}$ abdominal segment for RNAi in the abdomen. 1 µL of dsRNA solution was injected into the prepared hole. RNAi targeting the multiple genes was performed using an equivalent mixture of 100 µM dsRNA solutions (i.e., final concentration was 50 µM each for the RNAi of two genes).

Following the injection of the dsRNA solution, two droplets of LOGIQLEAN Gel for Ultrasound Hard type (GE Healthcare) were applied to the nymphal surface using forceps. The electrode (1 mmφ, NEPAGENE) was placed on the ultrasound gel, with a positive electrode on the side injected with the dsRNA solution and a negative electrode on the opposite side. 10-times electroporation pulses (280 ms/s each) were generated using the Cure-gene electroporator (CellProduce). In this study, 25 V was applied to *I. senegalensis* and 45 V to *P. zonata*. The treated nymphs were kept resting on a wet paper towel for approximately one day and transferred to a rearing case on the following day.

For *I. senegalensis*, the treated nymphs were kept individually in a petri dish (5 cm in diameter) containing approximately 10 mL of water and a piece of paper towel. As for antepenultimate or penultimate instar nymphs, nymphal molts into penultimate or final instar were observed in the petri dish.

As for RNAi of final instar nymphs, after the nymphs stop eating (when entering stage 3 of the final nymphal instar), move them individually into a plastic cage (10 cm × 15 cm, 3 cm water depth) with a disposable non-woven mesh for assisting adult emergence. For *P. zonata*, the treated nymphs were kept in a plastic cage (10 cm × 15 cm, 3 cm water depth) with a disposable non-woven mesh in a group of the same RNAi treatment.

After the nymphal ecdysis or adult emergence, the phenotype around the region where the positive electrode was placed for electroporation was observed and photographed using a stereoscopic microscope S8APO (Leica Microsystems) with a digital high definition microscope camera MC120HD (Leica) or a stereoscopic microscope S9D (Leica Microsystems) with a digital high definition microscope camera MC190HD (Leica). The molted nymphs and emerged adults were preserved in 100 % ethanol for future analyses. It should be noted that electroporation treatment was more lethal in the nymphs raised from eggs in the laboratory than the nymphs collected in the field (4). In order to eliminate this effect, the number of emerged (or molted) individuals is shown in the main text instead of the number of RNAi-treated individuals. Overall results of RNAi experiments were summarized in *SI Appendix,* Table S2.

**Phylogenetic analysis**

To construct the molecular phylogeny of zinc finger domains of *broad* gene, deduced amino-acid sequences were aligned using the Clustal W program implemented in MEGA 7 (version 7.0.26) (20). Zinc finger domains of *broad* gene of *Cloeon dipterum* were obtained by tBLASTn search ($P < 1e^{-10}$) (13) against the published genome of *Cloeon dipterum* (21). Molecular phylogenetic analyses were conducted by the neighbor-joining method and maximum likelihood method using MEGA 7 (20). Bootstrap values for neighbor-joining and maximum likelihood were obtained by 1,000 resampling.

**Scanning electron microscopy**

For scanning electron microscopy, nymphal and adult specimens preserved in 100% ethanol were dried in vacuo. Subsequently, the samples were gold-coated using Smart Coater (JEOL) and observed under a scanning electron microscope (JCM 6000, JEOL) operated at high vacuum and at 5 kV with use of the secondary electron signal.

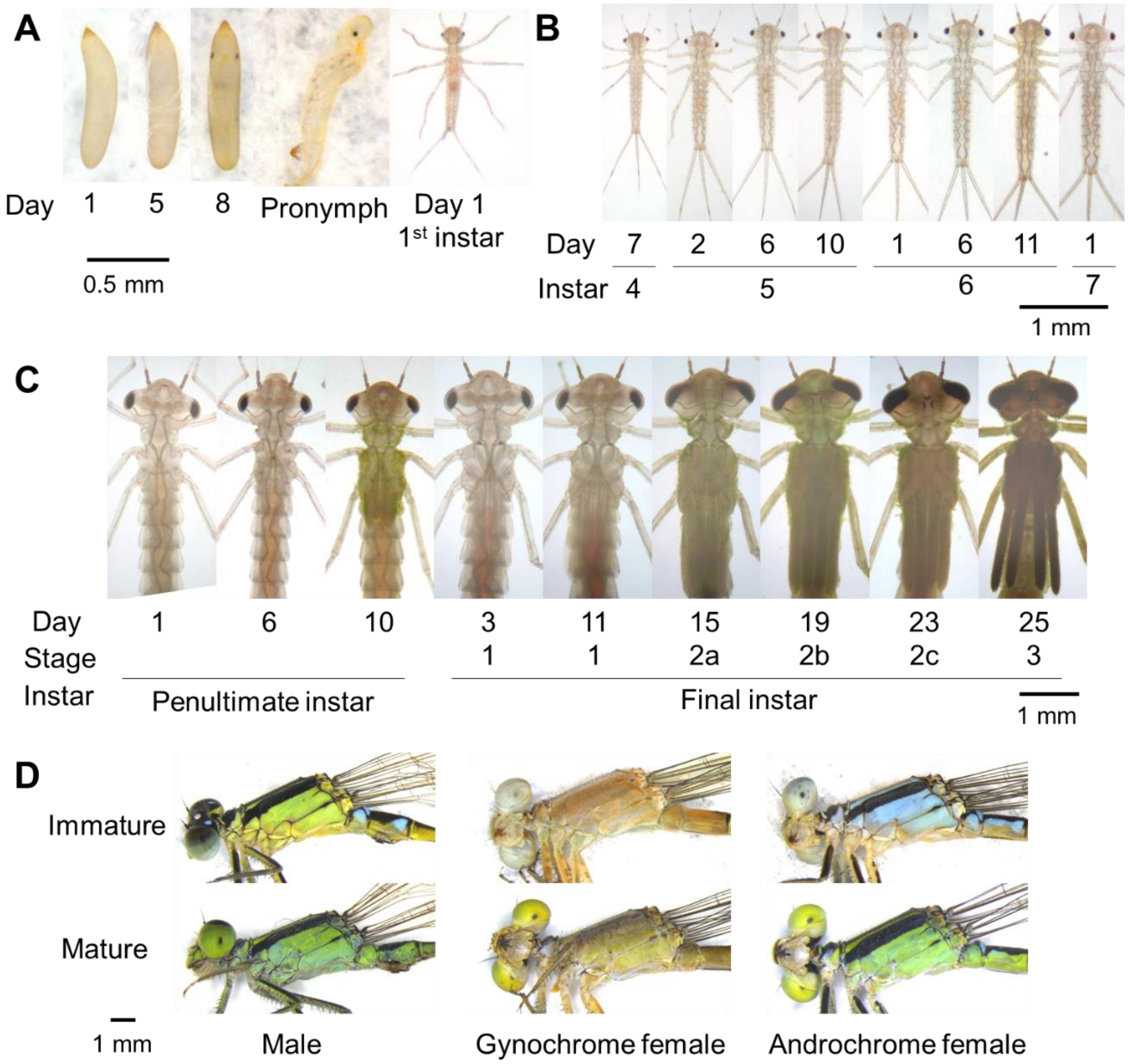


**Fig. S1.** Samples used for initial RNA sequencing. Sample information is listed in Table S1. (*A*) Eggs, pronymph, and 1st instar nymph. (*B*) Juvenile (from 4$^{th}$ to 7$^{th}$) instar nymphs. (*C*) Penultimate and final instar nymphs. (*D*) Immature (on the day of adult emergence) and mature adults of male, androchrome female, and gynochrome female.

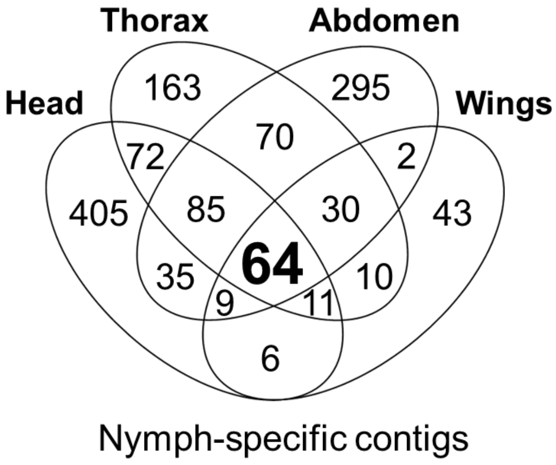


**Fig. S2.** The number of nymph-specific contigs and adult-specific contigs identified by the edgeR analysis (FDR value < 0.01).

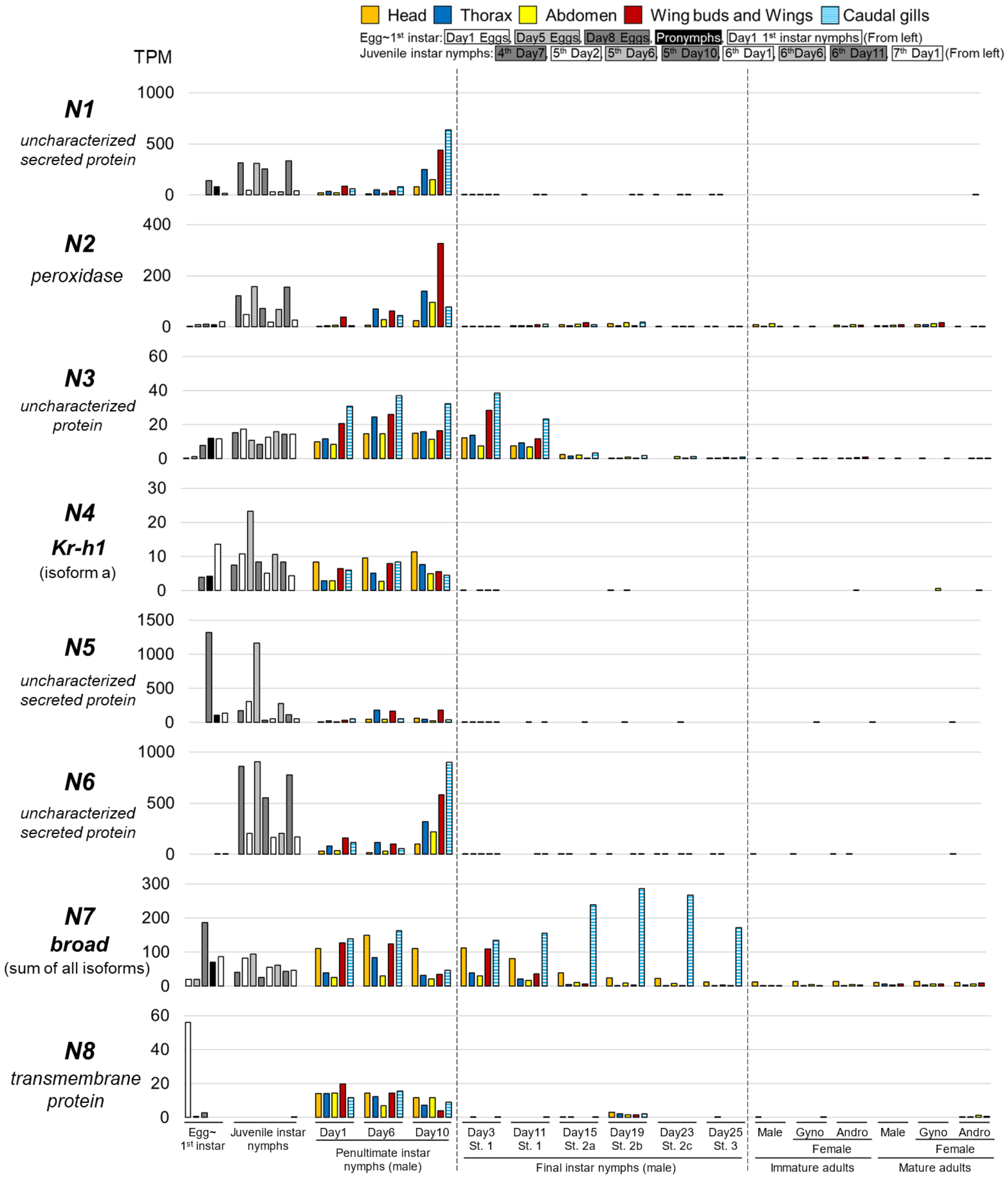


**Fig. S3.** Expression levels of 8 nymph-specific genes identified by the screening criteria in this study. Numbers on the Y axis indicate TPM values. Of the 8 nymph-specific genes, N3 and N7 (*broad*) genes were expressed in both the penultimate and final nymphal instar, while the other 6 genes were not highly expressed after the final nymphal instar.

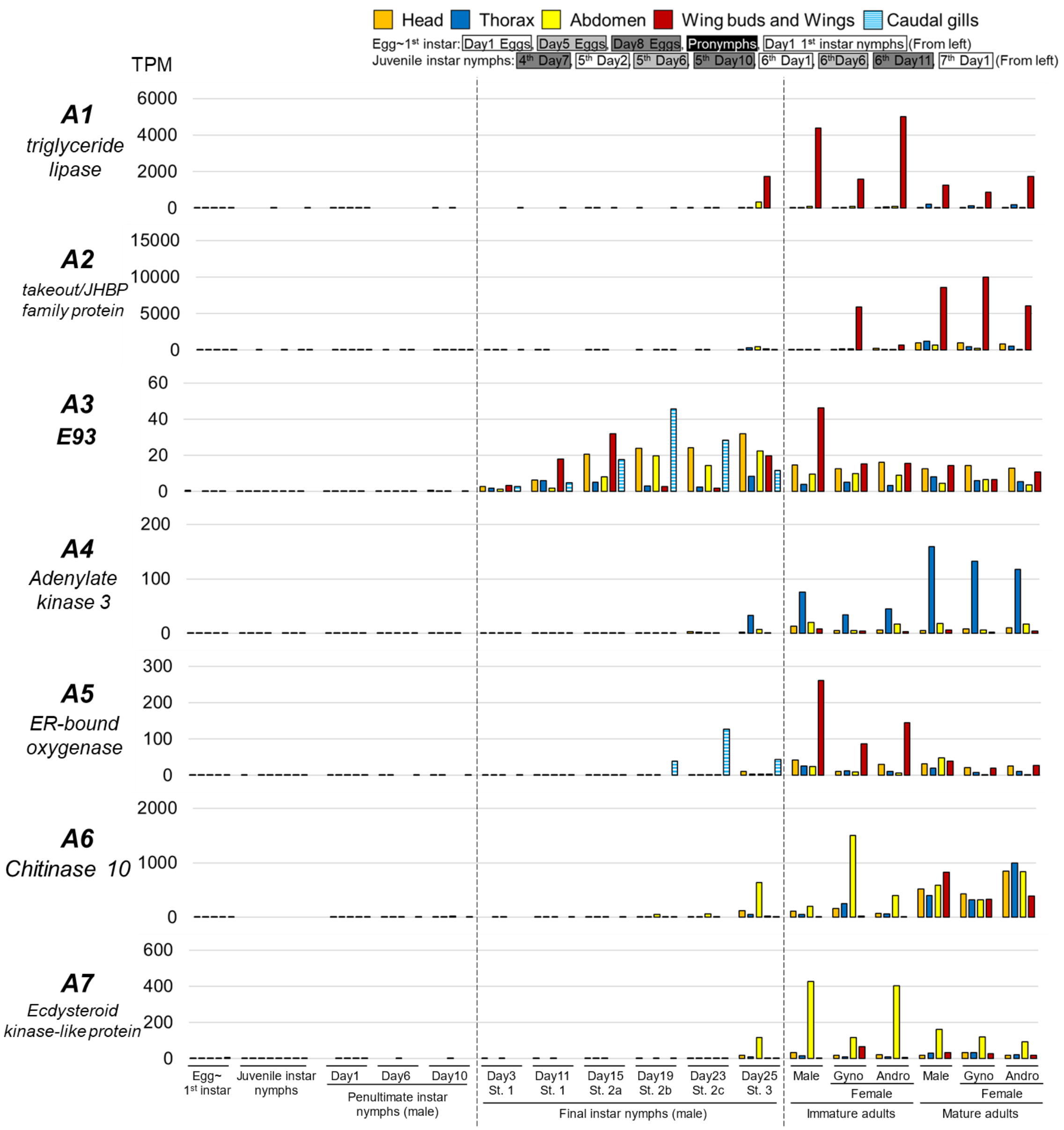


**Fig. S4.** Expression levels of 7 adult-specific genes identified by the screening criteria in this study. Numbers on the Y axis indicate TPM values. Expression of *E93*, A5, and the other 5 genes increased from day 11 (just prior to stage 2a), 19 (stage 2b), and 25 (stage 3), respectively.

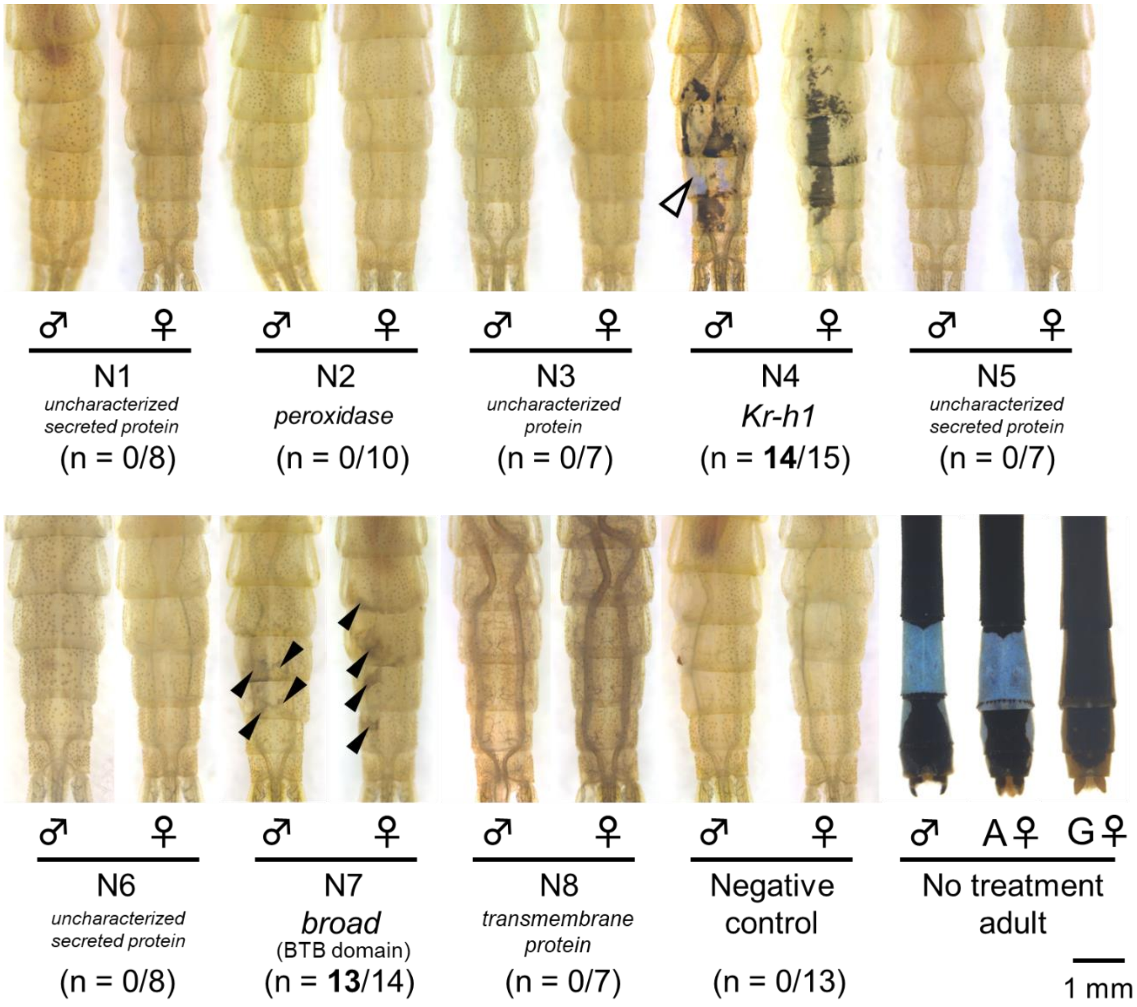


**Fig. S5.** RNAi phenotypes of 8 nymph-specific genes around the dorsal 8th abdominal segment of *I. senegalensis*. RNAi experiments were conducted at the early stage of the penultimate nymphal instar, and the phenotype was observed after ecdysis to the final instar. Numbers of parentheses indicate (number of individuals affected by RNAi / number of molted nymphs). White arrowhead indicates light-blue coloration, which is reminiscent of light-blue markings of adult males. Black arrowheads indicate the grayish regions. A♀: Androchrome female, G♀: Gynochrome female.

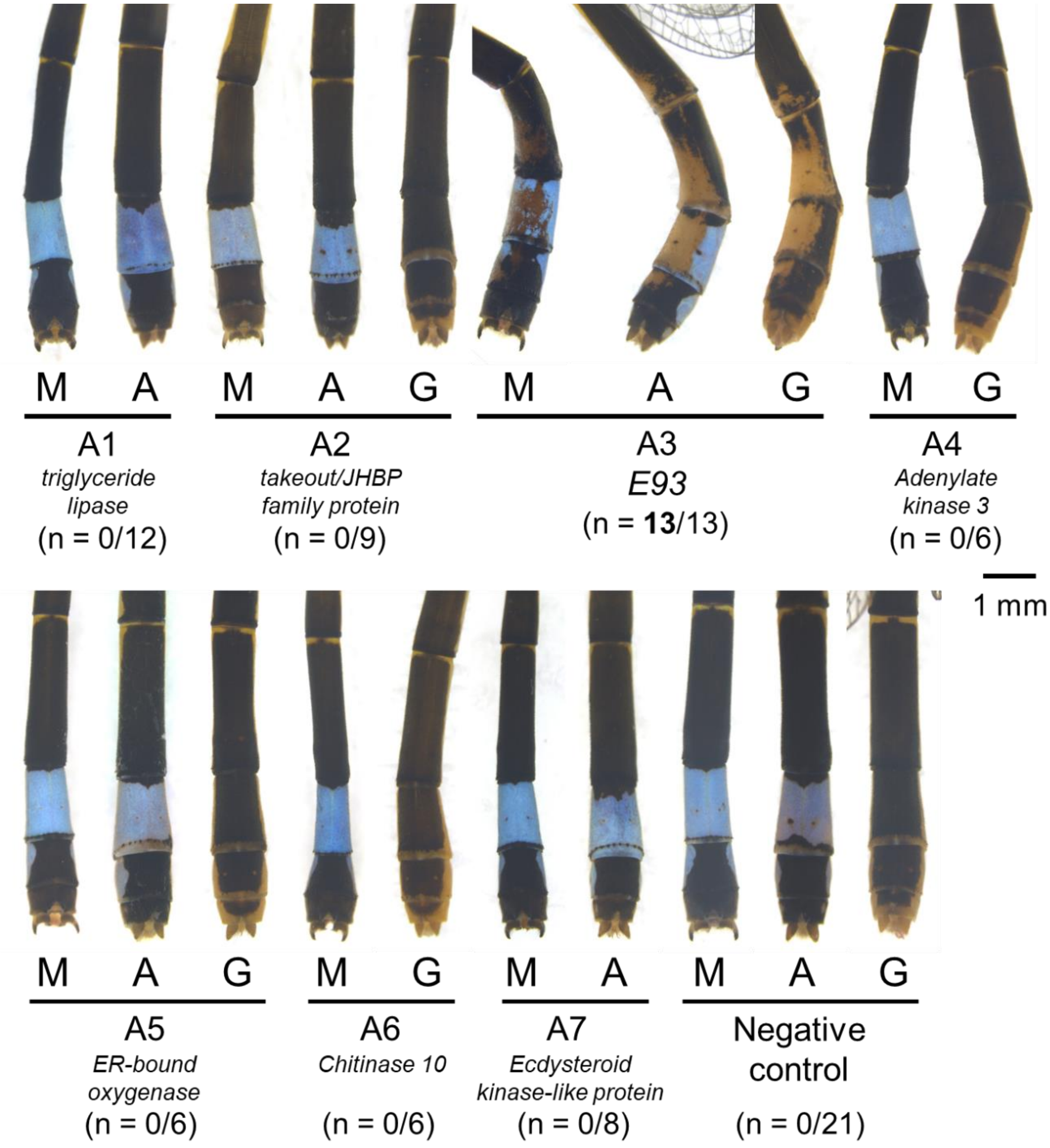


**Fig. S6.** RNAi phenotypes of 7 adult-specific genes around the dorsal 8$^{th}$ abdominal segment of *I. senegalensis*. Suppression of adult pigmentation was observed in *E93* RNAi individuals. RNAi experiments were conducted at stage 1 of the final nymphal instar, and RNAi phenotypes were observed after adult emergence. Numbers of parentheses indicate (number of individuals affected by RNAi / number of emerged adults). M: Male, A: Androchrome female, G: Gynochrome female.

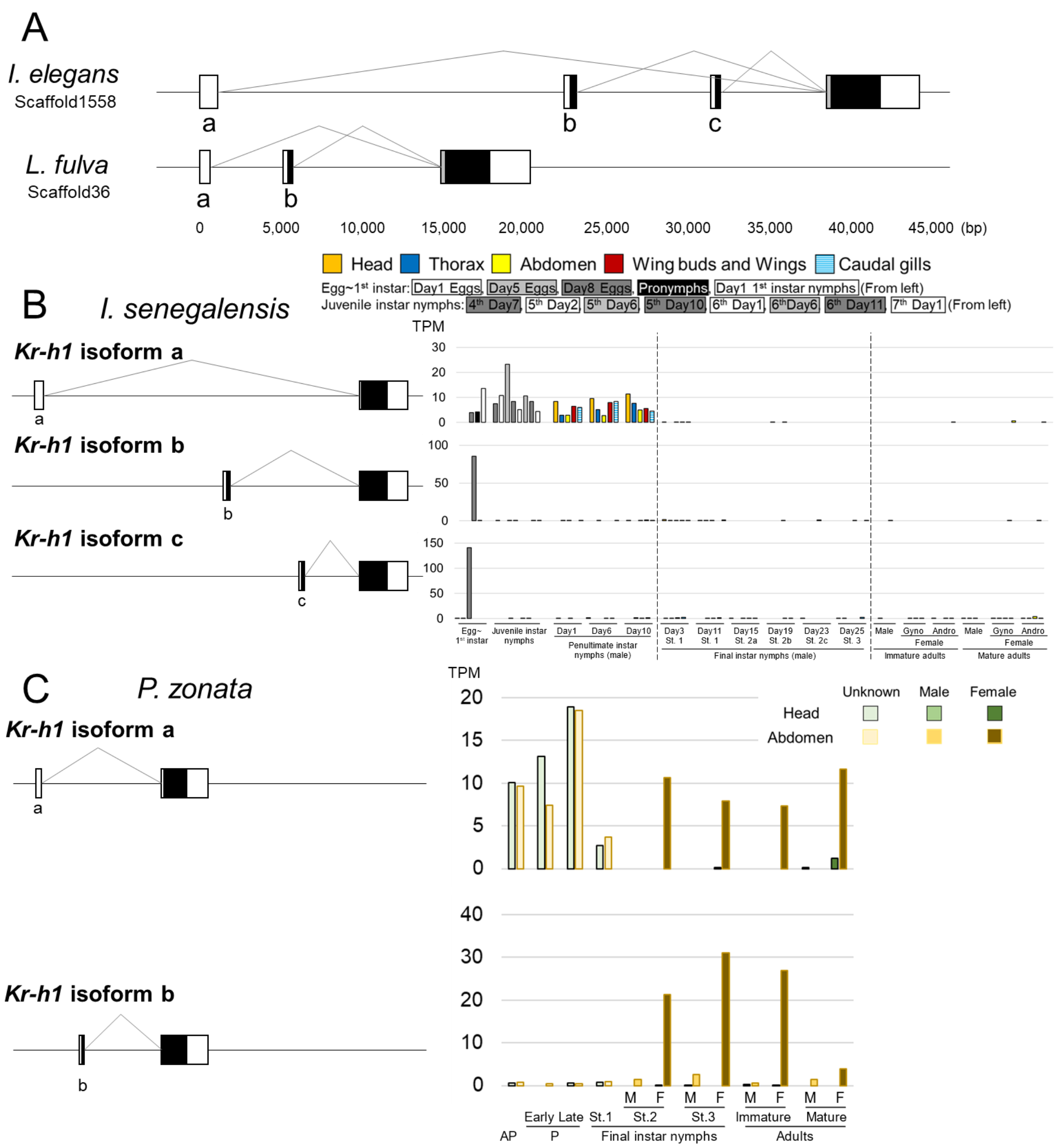


**Fig. S7.** Isoforms of *Kr-h1* gene in Odonata. (*A*) Gene structure of *Kr-h1* gene in the published genome of *Ischnura elegans* and *Ladona* (*Libellula*) *fulva*. Black boxes indicate coding sequence (CDS). (*B*) Expression levels of each isoform of *Kr-h1* gene in *I. senegalensis*. (C) Expression levels of each isoform of *Kr-h1* gene in *P. zonata*. In both species, only *Kr-h1* isoform a is expressed during penultimate nymphal instar. AP, P, M, and F indicate antepenultimate instar nymphs, penultimate instar nymphs, male, and female, respectively.

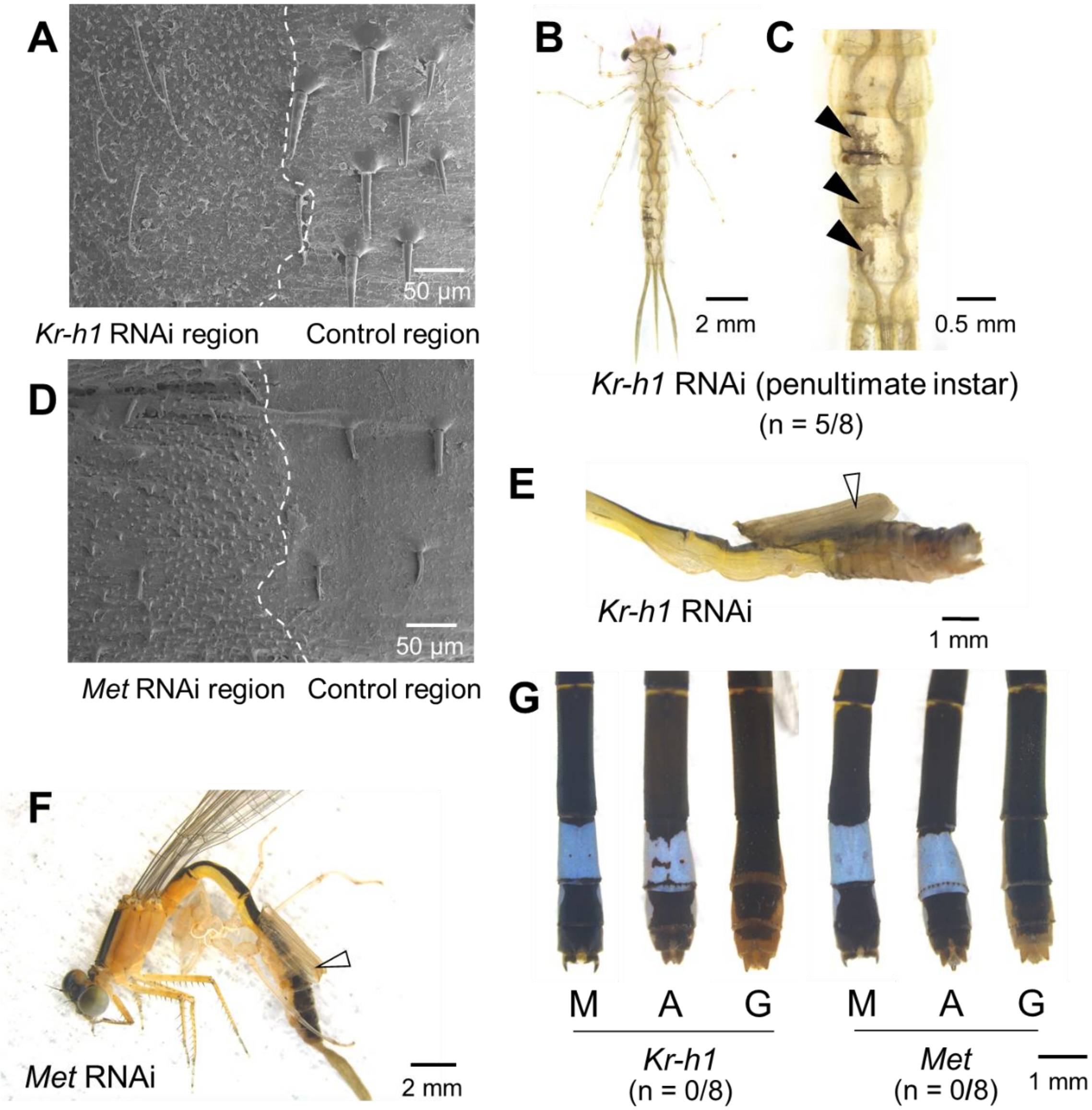


**Fig. S8.** *Kr-h1* RNAi and *Met* RNAi phenotypes. (*A*) SEM observation on the abdominal surface of *Kr-h1* RNAi individual. (*B*)(C) *Kr-h1* RNAi phenotype observed in the penultimate instar. RNAi was conducted at the antepenultimate instar. Black arrowheads indicate the RNAi phenotypes in which the adult-like surface structures and melanization were observed. (*C*) Magnified view around the RNAi region of (*B*). (*D*) SEM observation on the abdominal surface of *Met* RNAi individual. RNAi was conducted in the left side of the photo. (*E*)(*F*) RNAi phenotypes just after adult emergence. RNAi experiments were conducted at the early stage of the penultimate instar, and RNAi phenotypes were detected in the final instar. (*E*) *Kr-h1* RNAi. (*F*) *Met* RNAi. (*G*) *Kr-h1* RNAi and *Met* RNAi phenotype observed in adults. RNAi was conducted at stage 1 of the final nymphal instar. Numbers of parentheses indicate (number of individuals affected by RNAi / number of emerged adults or molted nymphs). M: Male, A: Androchrome female, G: Gynochrome female. White arrowheads indicate the exuviae bonded to the adult abdominal surface, due to the failure of ecdysis around RNAi region.

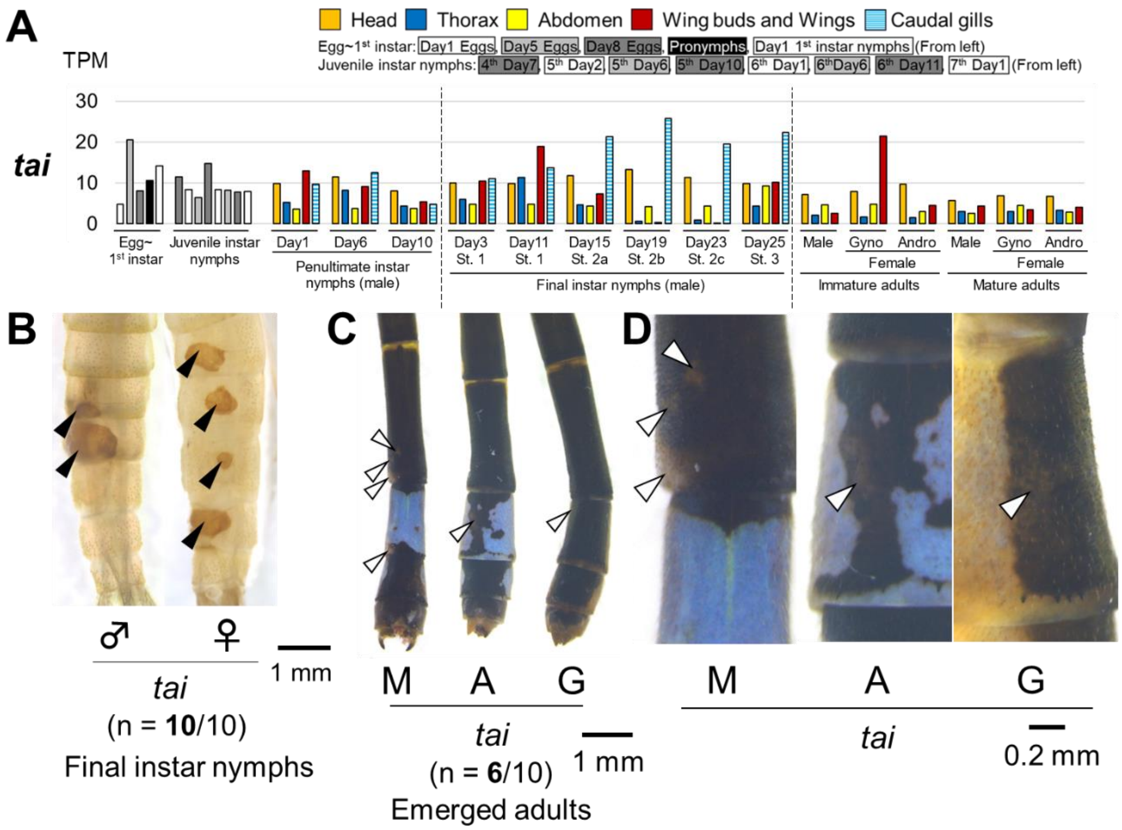


**Fig. S9.** Expression levels and RNAi phenotypes of *tai*. (*A*) Expression levels of *tai*. (*B*) RNAi phenotypes observed in the final instar nymphs. RNAi was conducted at the early stage of the penultimate instar. Black arrowheads indicate wound-like effects. (*C*) RNAi phenotypes observed in adults. RNAi was conducted at stage 1 of the final instar. (*D*) Magnified views of RNAi phenotype. White arrowheads indicate the inhibition of adult pigmentation. Numbers of parentheses indicate (number of individuals affected by RNAi / number of molted nymphs or emerged adults). M: Male, A: Androchrome female, G: Gynochrome female.

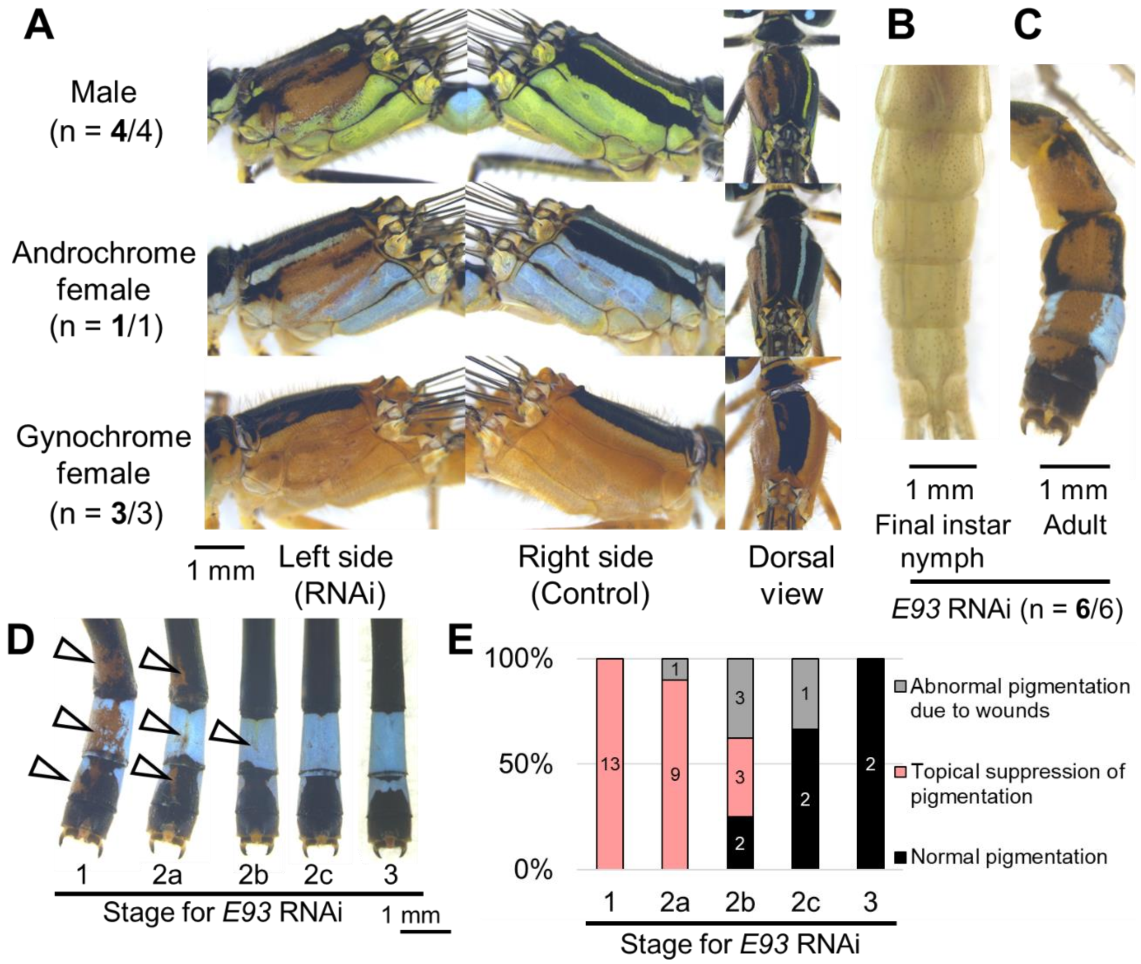


**Figure S10.** *E93* RNAi phenotypes in *I. senegalensis*. (*A*) RNAi phenotype in the thorax. RNAi was conducted in the left side of the thorax at stage 1 of the final nymphal instar. Numbers of parentheses indicate (number of individuals affected by RNAi / number of emerged adults). (*B*)(*C*) *E93* RNAi individual in which RNAi was conducted at the early stage of the penultimate instar. (*B*) The final instar nymph without significant RNAi effect. (*C*) RNAi phenotype observed in adult. (*B*) and (*C*) exhibit the same individual. (*D*) *E93* RNAi individuals in which RNAi was conducted at five morphological stages of the final instar. White arrowheads indicate the suppression of adult pigmentation. (*E*) The effect of RNAi at five morphological stages on adult pigmentation in *I. senegalensis*. The number on the bar indicates the number of individuals.

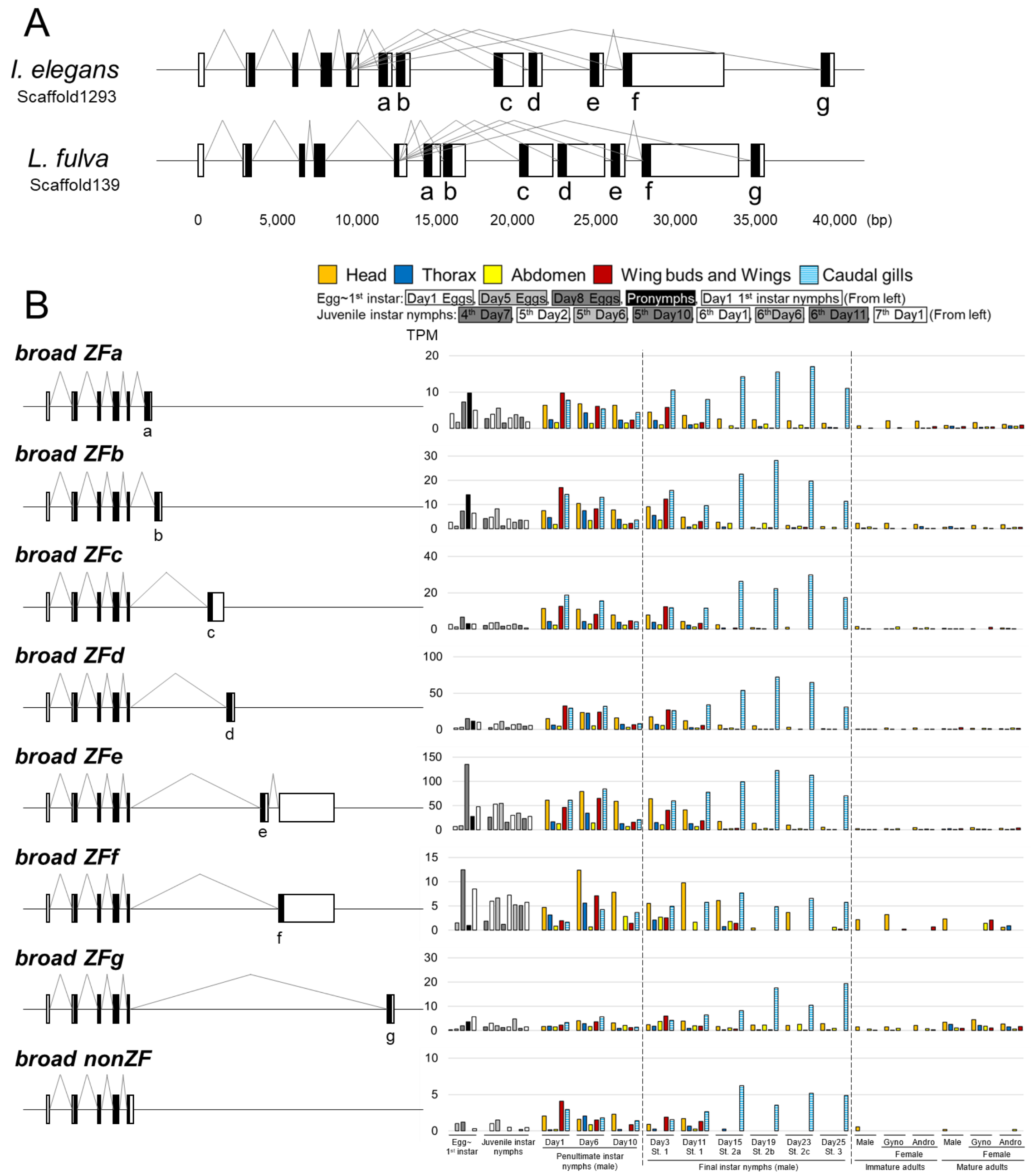


**Fig. S11.** Isoforms of *broad* gene in Odonata. (*A*) Gene structure of *broad* gene in the published genome of *I. elegans* and *L. fulva.* a, b, c, d, e, f, and g exons contain the zinc-finger domains, one of which are selected by alternative splicing. Black boxes indicate coding sequence (CDS). (*B*) Expression levels of each isoform of *broad* gene in *I. senegalensis*. In *I. senegalensis*, the expression patterns of the isoform were similar to each other, among which ZFd and ZFe exhibited relatively high expression levels.

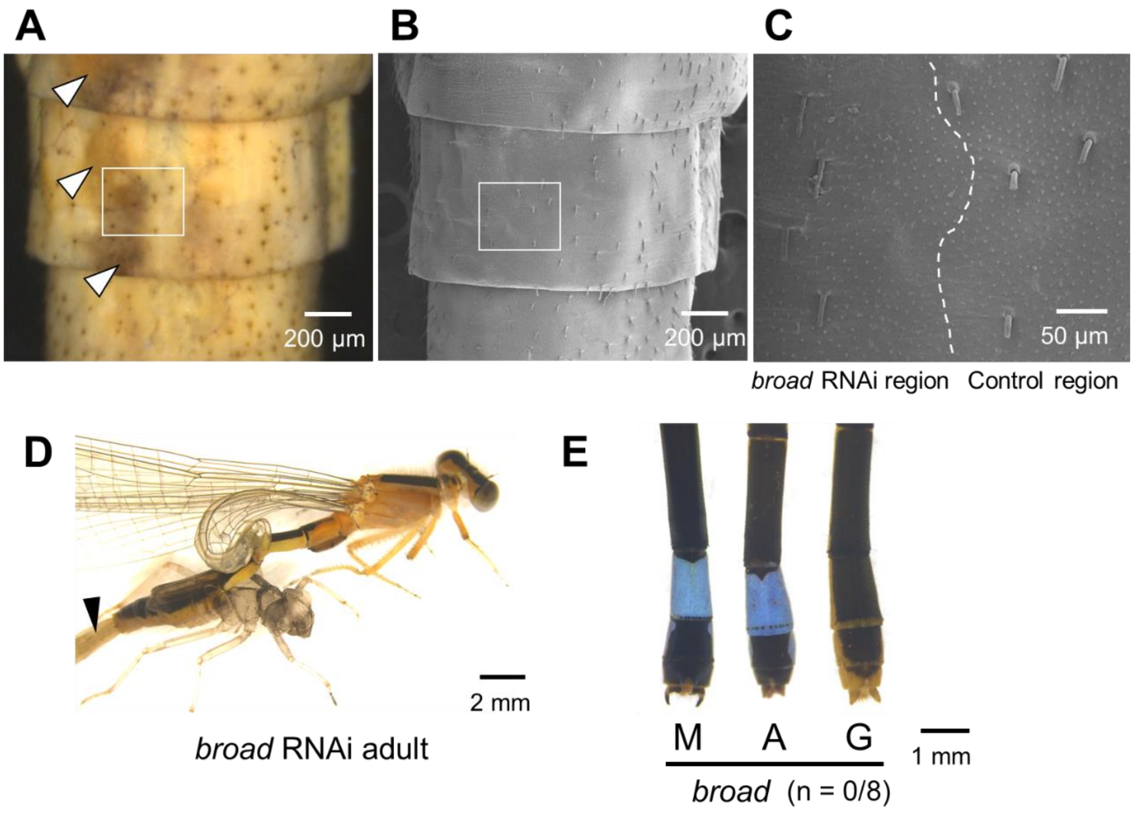


**Fig. S12.** RNAi phenotypes of *broad.* (*A*) Magnified view of *broad* RNAi region in dried specimen prepared for SEM observation. White arrowheads indicate grayish *broad* RNAi phenotypes. (*B*) SEM observation of abdominal surface. RNAi was conducted in the left side of the photo. No obvious difference in the surface structure was observed in the *broad* RNAi grayish region. It should be noted that the decrease of the number of bristles around the electroporation region was also observed in the *bla* (negative control) RNAi individuals, and the tip of bristles is easy to be accidentally broken. (*C*) A magnified view of boxed region in (*B*). (*A*), (*B*), and (*C*) exhibit the same individual. (*D*) *broad* RNAi phenotype just after adult emergence. RNAi experiments were conducted at the early stage of the penultimate instar, and RNAi phenotypes were detected in the final instar. Black arrowhead indicates the exuviae bonded to the adult abdominal surface, due to the failure of ecdysis around RNAi region. (*E*) RNAi phenotypes observed in adults. RNAi was conducted at stage 1 of the final instar. Male: Male, A: Androchrome female, G: Gynochrome female.

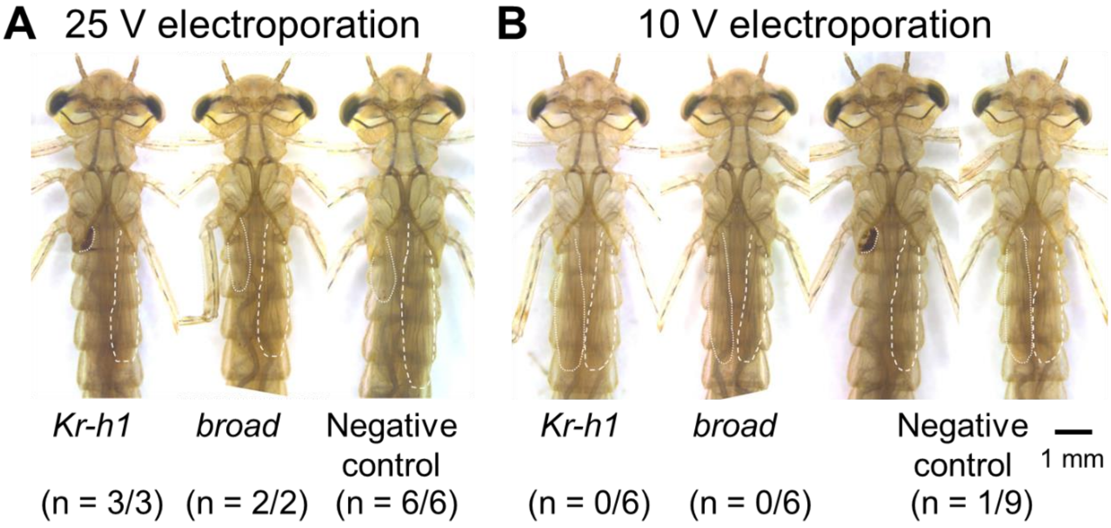


**Fig. S13.** RNAi phenotypes of *Kr-h1*, *broad*, and *bla* (negative control) in the wing bud (left side) of *I. senegalensis*. RNAi experiments were conducted at the early stage of the penultimate instar, and RNAi phenotypes were observed after ecdysis to the final instar. Numbers of parentheses indicate (number of individuals affected by RNAi / number of molted nymphs). White dotted line indicates the wing bud regions. (*A*) RNAi experiments conducted with 25 V electroporation. 25V electroporation itself damaged normal wing morphogenesis. (*B*) RNAi experiments conducted with 10V electroporation, in which no obvious effect was observed in most individuals.

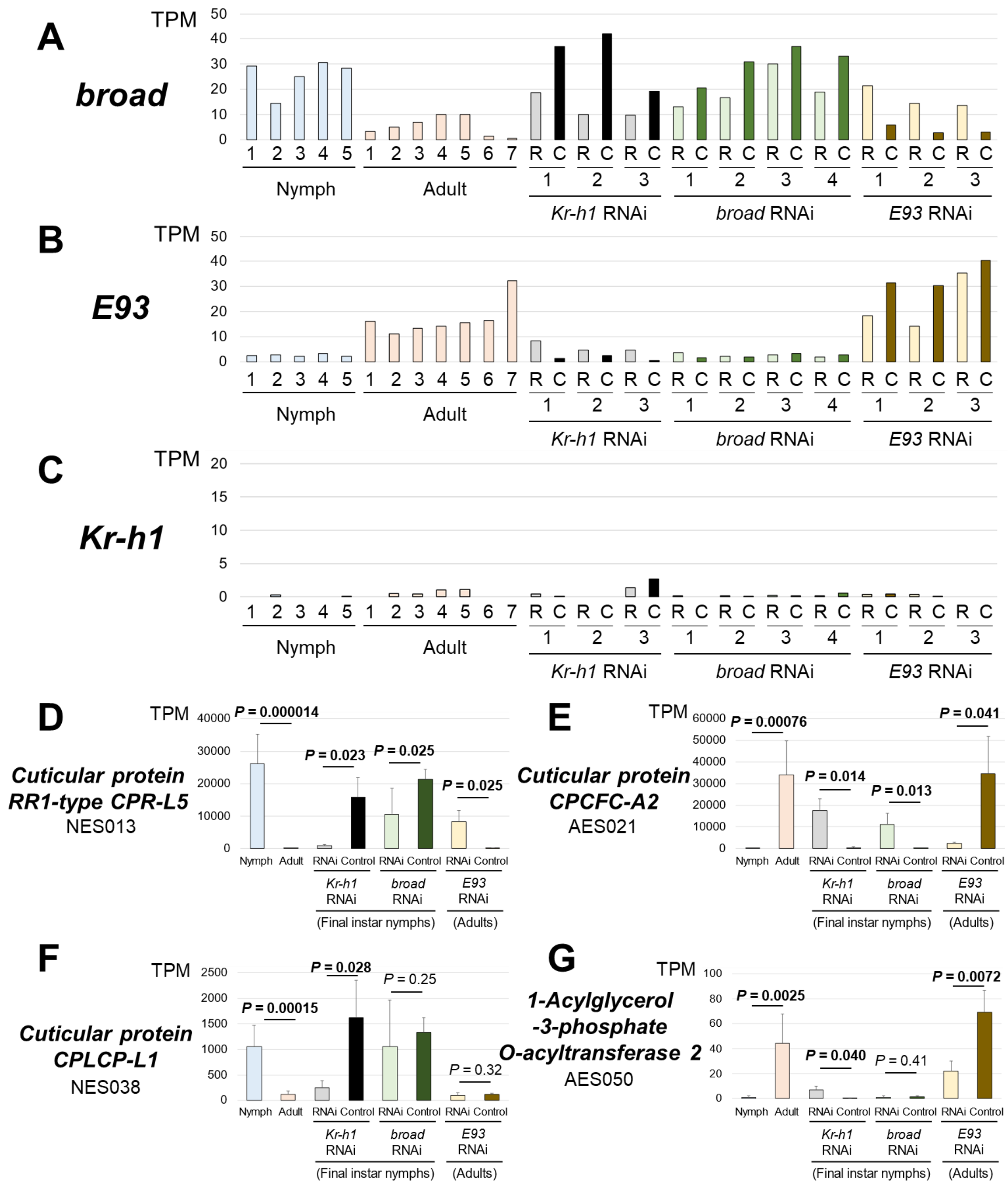


**Fig. S14.** Expression level changes after *Kr-h1*, *broad,* and *E93* RNAi treatments. (*A*) Expression levels of *broad* in each region and individual. (*B*) Expression levels of *E93* in each region and individual. (*C*) Expression levels of *Kr-h1* in each region and individual. R and C indicate RNAi region and control region, respectively. (*D*) *Cuticular protein RR1-type CPR-L5*, (*E*) *Cuticular protein CPCFC-A2*, (*F*) *Cuticular protein CPLCP-L1*, (*G*) *1-Acylglycerol-3-phosphate O-acyltransferase 2*. Numbers on the Y axis indicate TPM values. *P* value between untreated nymphs and adults was calculated by student *t*-test, while *P* value between RNAi regions and control regions was calculated by paired *t*-test. Error bars are SD. RNAi experiments were conducted at the early stage of the penultimate instar for *Kr-h1* RNAi (#1, #2) and *broad* RNAi, at the early stage of the antepenultimate instar for *Kr-h1* RNAi (#3), and at the early stage of final instar for *E93* RNAi. RNA was extracted after the following ecdysis.

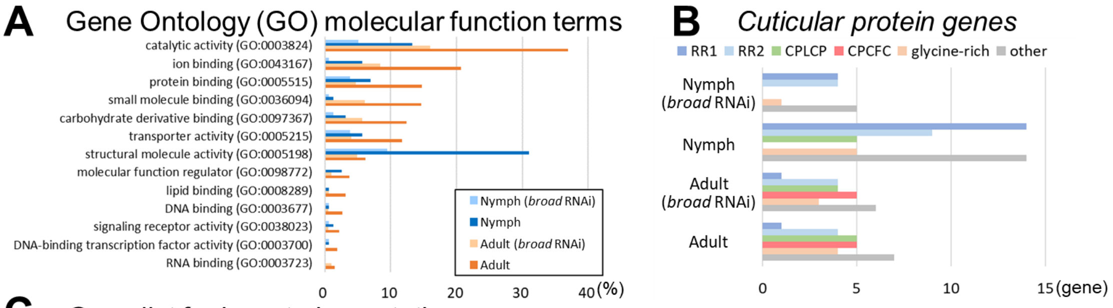


## C Gene list for insect pigmentation (based on Futahashi & Osanai-Futahashi, 2021[22])

| | Gene # | **nymphal/adult-epidermis-specific genes** (K: affected by *Kr-h1* RNAi, B: affect by *broad* RNAi, E: affected by *E93* RNAi ) |
|---|---|---|
| Nymph (Larva) | 0 | |
| Adult (melanin) | 4 | AES020: ***yellow-e1*** (KBE), AES040: ***MCO2*** (KBE), AES080: ***yellow-x1*** (E), AES088: ***DDC*** (KB) |
| (ommochrome) | 1 | AES065: ***cardinal*** (E) |
| (ommochrome, pteridine & uric acid) | 3 | AES241: ***os*** (KBE), AES250: ***white*** (KE), AES456: ***garnet*** (K) |
| (pteridine & uric acid) | 8 | AES011: ***Prat*** (KB), AES023: ***Pfas*** (KB), AES048: ***Paics*** (K), AES139: ***Gart*** (KB), AES158: ***raspberry*** (KBE), AES230: ***brown*** (E), AES235: ***purple*** (KE), AES522: ***oc*** (KE) |

## D Gene list for DNA-binding transcription factor activity (GO:0003700)

| | Gene # | **nymphal/adult-epidermis-specific genes** (K: affected by *Kr-h1* RNAi, B: affect by *broad* RNAi, E: affected by *E93* RNAi ) |
|---|---|---|
| Nymph (Larva) | 1 | LES099: ***broad*** (KBE) |
| Adult | 11 | AES239: ***E93*** (KE), AES342: ***ovo*** (E), AES347: ***buttonless*** (E), AES361: ***Pdp1*** (KE), AES370: ***Blimp-1*** (K), AES377: ***HR4*** (KE), AES457: ***CG4374*** (KBE), AES487: ***stc*** (-), AES488: ***kayak*** (K), AES498: ***datilografo*** (K), AES580: ***tango*** (KE) |

## E Gene list for signaling receptor (regulator) activity (GO:0038023, GO:0030545)

| | Gene # | **nymphal/adult-epidermis-specific genes** (K: affected by *Kr-h1* RNAi, B: affect by *broad* RNAi, E: affected by *E93* RNAi ) |
|---|---|---|
| Nymph (Larva) | 3 | LES004: ***GNBP3*** (KE), LES148: ***Dh44-R1*** (KB), LES149: ***hedgehog*** (-) |
| Adult | 16 | AES093: ***nAChRbeta3*** (KE), AES118: ***NtR1*** (K), AES195: ***orion*** (KE), AES201: ***torso-like*** (K), AES244: ***Cad96Ca*** (KE), AES259: ***AdipoR*** (KB), AES273: ***NtR2*** (KE), AES298: ***CrzR*** (KBE), AES319: ***argos*** (BE), AES359: ***Vha100-1*** (KE), AES362: ***EGFR*** (KE), AES377: ***HR4*** (KE), AES491: ***CCAP-R*** (KE), AES519: ***mthl15-1*** (KB), AES562: ***Ptp99A*** (E), AES573: ***mthl15-2*** (K) |

**Fig. S15.** Summary of nymphal/adult-epidermis-specific genes. Ratio of Gene Ontology (GO) molecular function terms (*A*) and number of cuticular protein genes (*B*) assigned to nymphal/adult-epidermis-specific genes. Genes affected *by broad* RNAi are also shown. Gene list for insect pigmentation (*C*), DNA-binding transcription factor activity (*D*), and signaling receptor (regulator) activity (*E*). Genes affected by RNAi of *Kr-h1*, *broad*, or *E93* are indicated in parenthesis by K, B, or E, respectively.

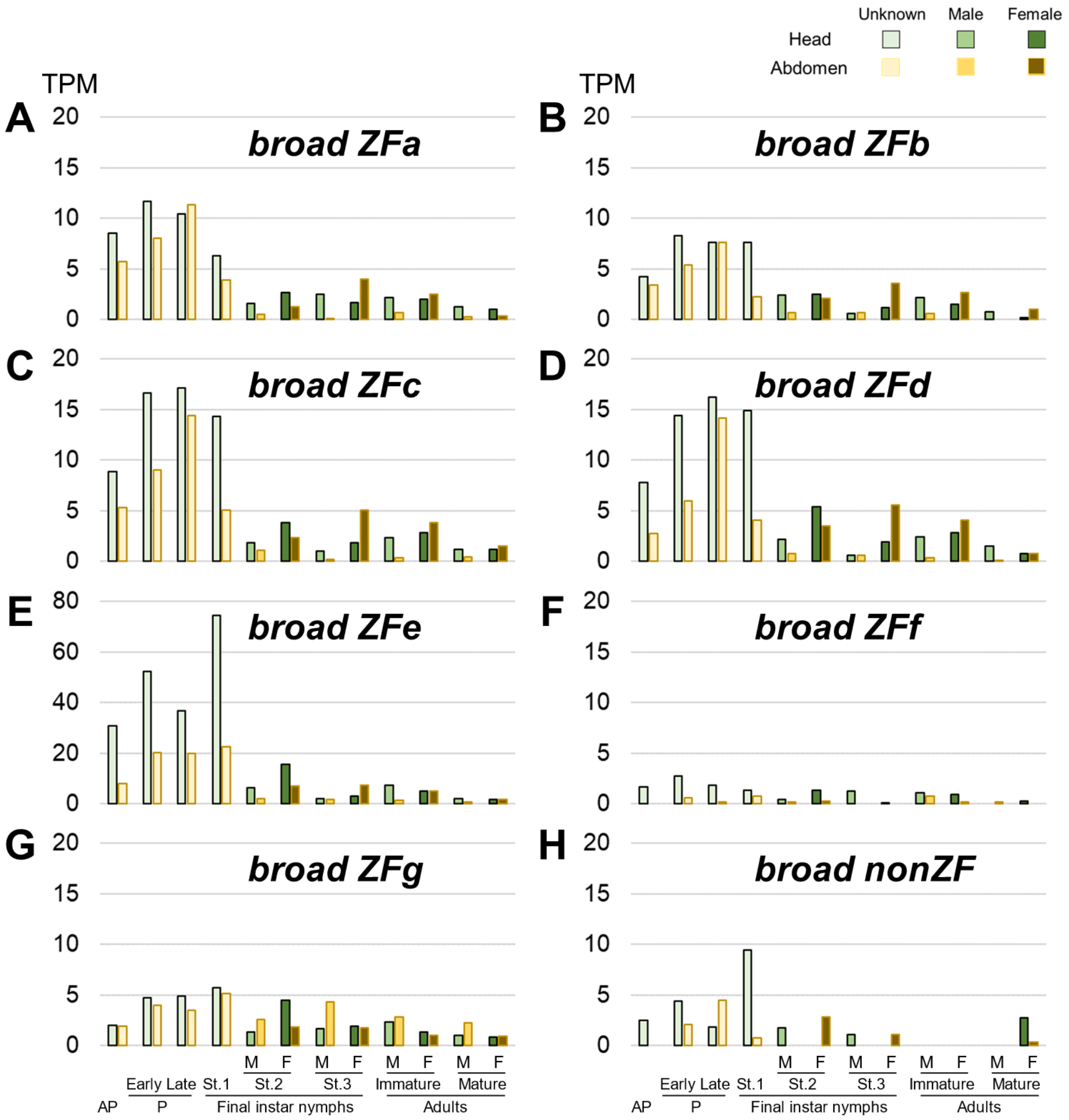


**Fig. S16.** Expression levels of *broad* isoforms in *P. zonata*. Numbers on the Y axis indicate TPM values. (*A*) ZFa, (*B*) ZFb, (*C*) ZFc, (*D*) ZFd, (*E*) ZFe, (*F*) ZFf, and (*G*) non ZF isoform. AP, P, M, and F indicate antepenultimate instar nymphs, penultimate instar nymphs, male, and female, respectively. Among seven isoforms of *P. zonata*, ZFa, ZFb, ZFc, ZFd, and ZFe were highly expressed during antepenultimate and penultimate nymphal instar, and ZFe exhibited relatively high expression levels.

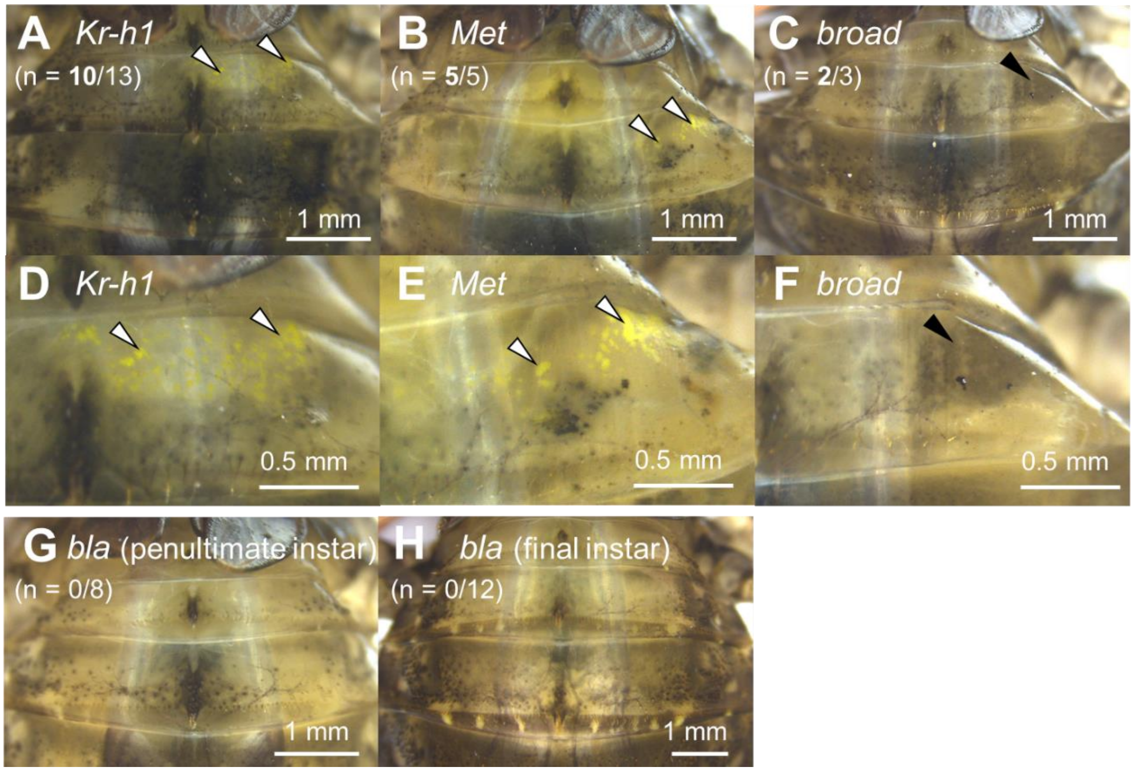


**Fig. S17.** RNAi phenotypes of *Kr-h1*, *Met*, *broad,* and *bla* (negative control) around the dorsal 4th abdominal segment of *P. zonata*. (*A*)-(*G*) RNAi experiments were conducted at the antepenultimate instar, and RNAi phenotypes were observed after ecdysis to the penultimate instar. (D), (E) and (F) are magnified views of (A), (B), and (C), respectively. (*A*)(*D*) *Kr-h1*, (*B*)(*E*) *Met*, (*C*)(*F*) *broad*, (*G*) *bla* (negative control). (*H*) *bla* (negative control) RNAi experiments were conducted final penultimate instar, and RNAi phenotypes were observed after ecdysis to the final instar. Numbers of parentheses indicate (number of individuals affected by RNAi / number of molted nymphs). White arrowheads indicate yellow markings, reminiscent of adult coloration. Black arrowheads indicate grayish region.

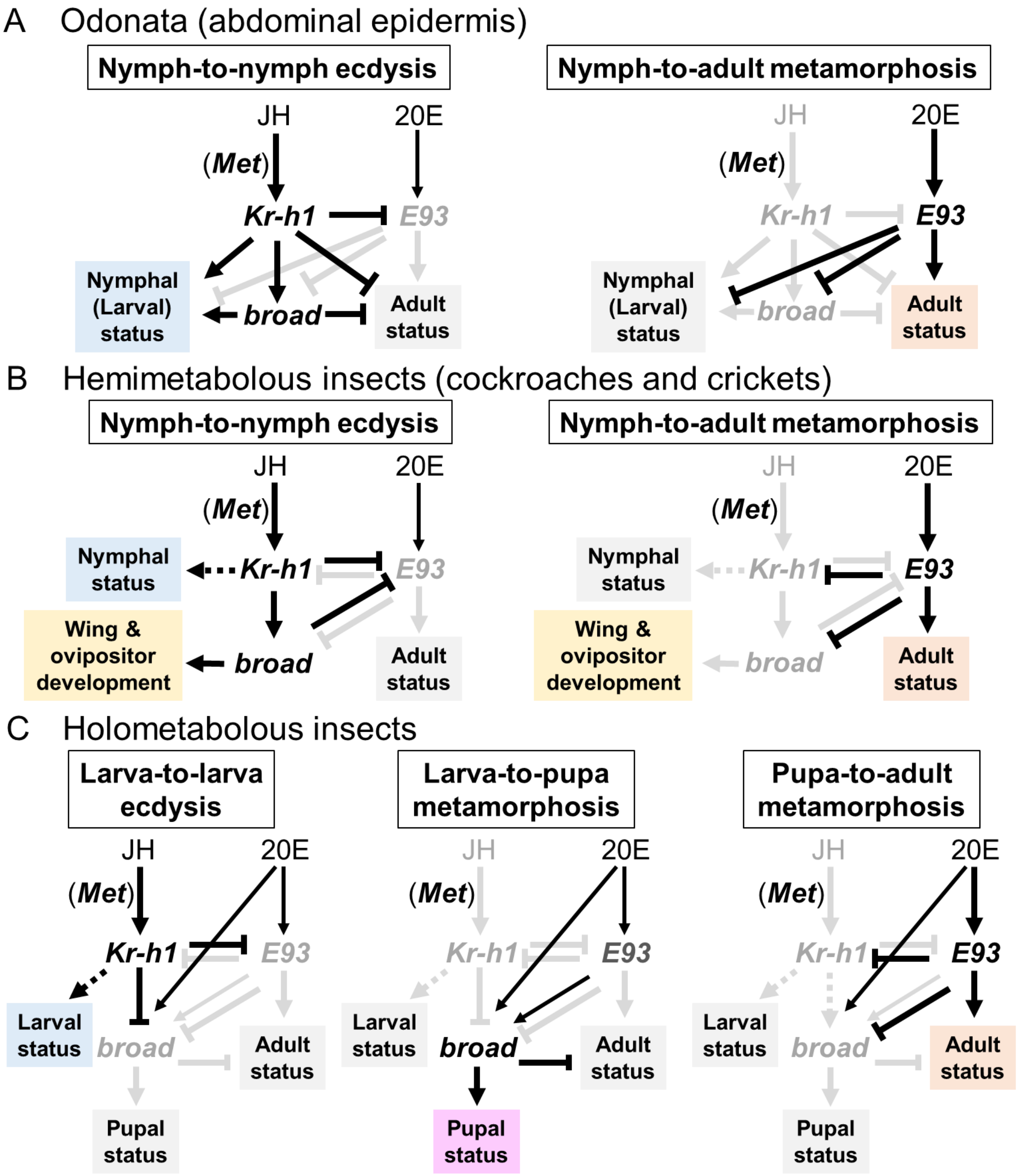


**Fig. S18.** Schematic summary of molecular mechanisms underlying metamorphosis in insects. (*A*) Odonata based on this study. (*B*) Other hemimetabolous insects. Figure is modified from Ureña et al. 2014[(23)], Ishimaru et al. 2019[(24)], and Belles 2020[(25)]. (*C*) Holometabolous insects. Figure is modified from Ureña et al. 2014[(23)], and Belles 2020[(25)]. In *Drosophila*, exogenous JH upregulates broad through Kr-h1 during pupa-to-adult metamorphosis (26). It should be noted that the interactions of three transcription factors of Odonata have been confirmed only in the abdominal epidermis.

**Table S1.** Sample and RNA-sequencing data used in this study.

| No. | Species | Individual | Sex | Region | Developmental stage | Date | No. of pairs | Sequence | Accession No. |
|---|---|---|---|---|---|---|---|---|---|
| 1 | *Ischnura senegalensis* | Mixture | U | Whole body | Day 1, 120 eggs | 2017.07.31 | 6,025,620 | HiSeq 100 bp paired | DRR278508 |
| 2 | *Ischnura senegalensis* | Mixture | U | Whole body | Day 5, 40 eggs | 2017.07.27 | 6,339,248 | HiSeq 100 bp paired | DRR278509 |
| 3 | *Ischnura senegalensis* | Mixture | U | Whole body | Day 8, 40 eggs | 2017.07.27 | 5,740,381 | HiSeq 100 bp paired | DRR278510 |
| 4 | *Ischnura senegalensis* | Mixture | U | Whole body | 3 Pronymphs | 2017.08.10 | 19,474,345 | HiSeq 150 bp paired | DRR278511 |
| 5 | *Ischnura senegalensis* | Mixture | U | Whole body | Day 1, 40 1st instar nymphs | 2017.06.04 | 5,887,185 | HiSeq 100 bp paired | DRR278512 |
| 6 | *Ischnura senegalensis* | Isen1 | U | Whole body | Day 7, 4th instar nymph | 2016.12.02 | 6,098,059 | HiSeq 100 bp paired | DRR278513 |
| 7 | *Ischnura senegalensis* | Isen2 | U | Whole body | Day 2, 5th instar nymph | 2016.12.03 | 6,237,627 | HiSeq 100 bp paired | DRR278514 |
| 8 | *Ischnura senegalensis* | Isen3 | U | Whole body | Day 6, 5th instar nymph | 2016.12.04 | 6,860,496 | HiSeq 100 bp paired | DRR278515 |
| 9 | *Ischnura senegalensis* | Isen4 | U | Whole body | Day 10, 5th instar nymph | 2016.12.09 | 6,753,981 | HiSeq 100 bp paired | DRR278516 |
| 10 | *Ischnura senegalensis* | Isen5 | U | Whole body | Day 1, 6th instar nymph | 2016.12.09 | 6,419,895 | HiSeq 100 bp paired | DRR278517 |
| 11 | *Ischnura senegalensis* | Isen6 | U | Whole body | Day 6, 6th instar nymph | 2016.12.12 | 6,809,773 | HiSeq 100 bp paired | DRR278518 |
| 12 | *Ischnura senegalensis* | Isen7 | U | Whole body | Day 11, 6th instar nymph | 2016.12.18 | 7,609,554 | HiSeq 100 bp paired | DRR278519 |
| 13 | *Ischnura senegalensis* | Isen8 | U | Whole body | Day 1, 7th instar nymph | 2016.12.19 | 7,085,226 | HiSeq 100 bp paired | DRR278520 |
| 14 | *Ischnura senegalensis* | Isen9 | M | Head | Day 1, penultimate instar nymph | 2017.11.06 | 12,576,950 | HiSeq 100 bp paired | DRR278521 |
| 15 | *Ischnura senegalensis* | Isen9 | M | Thorax | Day 1, penultimate instar nymph | 2017.11.06 | 14,058,608 | HiSeq 100 bp paired | DRR278522 |
| 16 | *Ischnura senegalensis* | Isen9 | M | Abdomen | Day 1, penultimate instar nymph | 2017.11.06 | 13,281,142 | HiSeq 100 bp paired | DRR278523 |
| 17 | *Ischnura senegalensis* | Isen9 | M | Wing buds | Day 1, penultimate instar nymph | 2017.11.06 | 12,383,636 | HiSeq 100 bp paired | DRR278524 |
| 18 | *Ischnura senegalensis* | Isen9 | M | Caudal gills | Day 1, penultimate instar nymph | 2017.11.06 | 13,722,536 | HiSeq 100 bp paired | DRR278525 |
| 19 | *Ischnura senegalensis* | Isen10 | M | Head | Day 6, penultimate instar nymph | 2017.02.06 | 6,175,552 | HiSeq 100 bp paired | DRR278526 |
| 20 | *Ischnura senegalensis* | Isen10 | M | Thorax | Day 6, penultimate instar nymph | 2017.02.06 | 7,082,167 | HiSeq 100 bp paired | DRR278527 |
| 21 | *Ischnura senegalensis* | Isen10 | M | Abdomen | Day 6, penultimate instar nymph | 2017.02.06 | 7,291,344 | HiSeq 100 bp paired | DRR278528 |
| 22 | *Ischnura senegalensis* | Isen10 | M | Wing buds | Day 6, penultimate instar nymph | 2017.02.06 | 4,927,711 | HiSeq 100 bp paired | DRR278529 |
| 23 | *Ischnura senegalensis* | Isen10 | M | Caudal gills | Day 6, penultimate instar nymph | 2017.02.06 | 6,530,420 | HiSeq 100 bp paired | DRR278530 |
| 24 | *Ischnura senegalensis* | Isen11 | M | Head | Day 10, penultimate instar nymph | 2017.02.06 | 6,775,645 | HiSeq 100 bp paired | DRR278531 |
| 25 | *Ischnura senegalensis* | Isen11 | M | Thorax | Day 10, penultimate instar nymph | 2017.02.06 | 6,625,542 | HiSeq 100 bp paired | DRR278532 |
| 26 | *Ischnura senegalensis* | Isen11 | M | Abdomen | Day 10, penultimate instar nymph | 2017.02.06 | 6,979,181 | HiSeq 100 bp paired | DRR278533 |
| 27 | *Ischnura senegalensis* | Isen11 | M | Wing buds | Day 10, penultimate instar nymph | 2017.02.06 | 8,277,848 | HiSeq 100 bp paired | DRR278534 |
| 28 | *Ischnura senegalensis* | Isen11 | M | Caudal gills | Day 10, penultimate instar nymph | 2017.02.06 | 7,409,469 | HiSeq 100 bp paired | DRR278535 |
| 29 | *Ischnura senegalensis* | Isen12 | M | Head | Day 3, final instar nymph | 2016.12.27 | 7,021,307 | HiSeq 100 bp paired | DRR278536 |
| 30 | *Ischnura senegalensis* | Isen12 | M | Thorax | Day 3, final instar nymph | 2016.12.27 | 6,097,757 | HiSeq 100 bp paired | DRR278537 |
| 31 | *Ischnura senegalensis* | Isen12 | M | Abdomen | Day 3, final instar nymph | 2016.12.27 | 6,546,475 | HiSeq 100 bp paired | DRR278538 |
| 32 | *Ischnura senegalensis* | Isen12 | M | Wing buds | Day 3, final instar nymph | 2016.12.27 | 7,031,172 | HiSeq 100 bp paired | DRR278539 |
| 33 | *Ischnura senegalensis* | Isen12 | M | Caudal gills | Day 3, final instar nymph | 2016.12.27 | 7,375,684 | HiSeq 100 bp paired | DRR278540 |
| 34 | *Ischnura senegalensis* | Isen13 | M | Head | Day 11, final instar nymph | 2016.12.26 | 5,656,713 | HiSeq 100 bp paired | DRR278541 |
| 35 | *Ischnura senegalensis* | Isen13 | M | Thorax | Day 11, final instar nymph | 2016.12.26 | 6,380,056 | HiSeq 100 bp paired | DRR278542 |
| 36 | *Ischnura senegalensis* | Isen13 | M | Abdomen | Day 11, final instar nymph | 2016.12.26 | 7,894,950 | HiSeq 100 bp paired | DRR278543 |
| 37 | *Ischnura senegalensis* | Isen13 | M | Wing buds | Day 11, final instar nymph | 2016.12.26 | 7,131,924 | HiSeq 100 bp paired | DRR278544 |
| 38 | *Ischnura senegalensis* | Isen13 | M | Caudal gills | Day 11, final instar nymph | 2016.12.26 | 5,249,082 | HiSeq 100 bp paired | DRR278545 |
| 39 | *Ischnura senegalensis* | Isen14 | M | Head | Day 15, final instar nymph | 2017.01.10 | 7,216,078 | HiSeq 100 bp paired | DRR278546 |
| 40 | *Ischnura senegalensis* | Isen14 | M | Thorax | Day 15, final instar nymph | 2017.01.10 | 6,803,229 | HiSeq 100 bp paired | DRR278547 |
| 41 | *Ischnura senegalensis* | Isen14 | M | Abdomen | Day 15, final instar nymph | 2017.01.10 | 6,210,599 | HiSeq 100 bp paired | DRR278548 |
| 42 | *Ischnura senegalensis* | Isen14 | M | Wing buds | Day 15, final instar nymph | 2017.01.10 | 4,967,228 | HiSeq 100 bp paired | DRR278549 |
| 43 | *Ischnura senegalensis* | Isen14 | M | Caudal gills | Day 15, final instar nymph | 2017.01.10 | 6,239,575 | HiSeq 100 bp paired | DRR278550 |
| 44 | *Ischnura senegalensis* | Isen15 | M | Head | Day 19, final instar nymph | 2016.12.30 | 5,644,182 | HiSeq 100 bp paired | DRR278551 |
| 45 | *Ischnura senegalensis* | Isen15 | M | Thorax | Day 19, final instar nymph | 2016.12.30 | 8,095,303 | HiSeq 100 bp paired | DRR278552 |
| 46 | *Ischnura senegalensis* | Isen15 | M | Abdomen | Day 19, final instar nymph | 2016.12.30 | 5,826,374 | HiSeq 100 bp paired | DRR278553 |
| 47 | *Ischnura senegalensis* | Isen15 | M | Wing buds | Day 19, final instar nymph | 2016.12.30 | 6,403,970 | HiSeq 100 bp paired | DRR278554 |
| 48 | *Ischnura senegalensis* | Isen15 | M | Caudal gills | Day 19, final instar nymph | 2016.12.30 | 3,429,068 | HiSeq 100 bp paired | DRR278555 |
| 49 | *Ischnura senegalensis* | Isen16 | M | Head | Day 23, final instar nymph | 2017.01.03 | 5,948,406 | HiSeq 100 bp paired | DRR278556 |
| 50 | *Ischnura senegalensis* | Isen16 | M | Thorax | Day 23, final instar nymph | 2017.01.03 | 5,240,332 | HiSeq 100 bp paired | DRR278557 |
| 51 | *Ischnura senegalensis* | Isen16 | M | Abdomen | Day 23, final instar nymph | 2017.01.03 | 5,865,360 | HiSeq 100 bp paired | DRR278558 |
| 52 | *Ischnura senegalensis* | Isen16 | M | Wing buds | Day 23, final instar nymph | 2017.01.03 | 6,412,838 | HiSeq 100 bp paired | DRR278559 |
| 53 | *Ischnura senegalensis* | Isen16 | M | Caudal gills | Day 23, final instar nymph | 2017.01.03 | 4,190,630 | HiSeq 100 bp paired | DRR278560 |
| 54 | *Ischnura senegalensis* | Isen17 | M | Head | Day 25, final instar nymph | 2017.01.06 | 5,985,070 | HiSeq 100 bp paired | DRR278561 |
| 55 | *Ischnura senegalensis* | Isen17 | M | Thorax | Day 25, final instar nymph | 2017.01.06 | 5,943,137 | HiSeq 100 bp paired | DRR278562 |
| 56 | *Ischnura senegalensis* | Isen17 | M | Abdomen | Day 25, final instar nymph | 2017.01.06 | 5,390,833 | HiSeq 100 bp paired | DRR278563 |
| 57 | *Ischnura senegalensis* | Isen17 | M | Wing buds | Day 25, final instar nymph | 2017.01.06 | 5,704,902 | HiSeq 100 bp paired | DRR278564 |
| 58 | *Ischnura senegalensis* | Isen17 | M | Caudal gills | Day 25, final instar nymph | 2017.01.06 | 3,468,078 | HiSeq 100 bp paired | DRR278565 |
| 59 | *Ischnura senegalensis* | Isen18 | M | Head | Day 1, immature adult | 2017.05.07 | 6,522,226 | HiSeq 100 bp paired | DRR278566 |
| 60 | *Ischnura senegalensis* | Isen18 | M | Thorax | Day 1, immature adult | 2017.05.07 | 5,827,779 | HiSeq 100 bp paired | DRR278567 |
| 61 | *Ischnura senegalensis* | Isen18 | M | Abdomen | Day 1, immature adult | 2017.05.07 | 5,931,828 | HiSeq 100 bp paired | DRR278568 |
| 62 | *Ischnura senegalensis* | Isen18 | M | Wings | Day 1, immature adult | 2017.05.07 | 6,791,080 | HiSeq 100 bp paired | DRR278569 |
| 63 | *Ischnura senegalensis* | Isen19 | GF | Head | Day 1, immature adult | 2017.05.12 | 5,956,338 | HiSeq 100 bp paired | DRR278570 |
| 64 | *Ischnura senegalensis* | Isen19 | GF | Thorax | Day 1, immature adult | 2017.05.12 | 7,050,676 | HiSeq 100 bp paired | DRR278571 |

| | | | | | | | | | |
|---|---|---|---|---|---|---|---|---|---|
| 65 | *Ischnura senegalensis* | Isen19 | GF | Abdomen | Day 1, immature adult | 2017.05.12 | 5,078,891 | HiSeq 100 bp paired | DRR278572 |
| 66 | *Ischnura senegalensis* | Isen19 | GF | Wings | Day 1, immature adult | 2017.05.12 | 6,353,827 | HiSeq 100 bp paired | DRR278573 |
| 67 | *Ischnura senegalensis* | Isen20 | AF | Head | Day 1, immature adult | 2017.05.12 | 6,551,070 | HiSeq 100 bp paired | DRR278574 |
| 68 | *Ischnura senegalensis* | Isen20 | AF | Thorax | Day 1, immature adult | 2017.05.12 | 6,940,116 | HiSeq 100 bp paired | DRR278575 |
| 69 | *Ischnura senegalensis* | Isen20 | AF | Abdomen | Day 1, immature adult | 2017.05.12 | 7,652,578 | HiSeq 100 bp paired | DRR278576 |
| 70 | *Ischnura senegalensis* | Isen20 | AF | Wings | Day 1, immature adult | 2017.05.12 | 5,954,785 | HiSeq 100 bp paired | DRR278577 |
| 71 | *Ischnura senegalensis* | Isen21 | M | Head | Mature adult | 2017.07.24 | 6,861,939 | HiSeq 100 bp paired | DRR278578 |
| 72 | *Ischnura senegalensis* | Isen21 | M | Thorax | Mature adult | 2017.07.24 | 9,694,424 | HiSeq 100 bp paired | DRR278579 |
| 73 | *Ischnura senegalensis* | Isen21 | M | Abdomen | Mature adult | 2017.07.24 | 6,183,962 | HiSeq 100 bp paired | DRR278580 |
| 74 | *Ischnura senegalensis* | Isen21 | M | Wings | Mature adult | 2017.07.24 | 5,041,979 | HiSeq 100 bp paired | DRR278581 |
| 75 | *Ischnura senegalensis* | Isen22 | GF | Head | Mature adult | 2017.07.24 | 6,239,759 | HiSeq 100 bp paired | DRR278582 |
| 76 | *Ischnura senegalensis* | Isen22 | GF | Thorax | Mature adult | 2017.07.24 | 4,633,932 | HiSeq 100 bp paired | DRR278583 |
| 77 | *Ischnura senegalensis* | Isen22 | GF | Abdomen | Mature adult | 2017.07.24 | 7,420,696 | HiSeq 100 bp paired | DRR278584 |
| 78 | *Ischnura senegalensis* | Isen22 | GF | Wings | Mature adult | 2017.07.24 | 5,688,283 | HiSeq 100 bp paired | DRR278585 |
| 79 | *Ischnura senegalensis* | Isen23 | AF | Head | Mature adult | 2017.07.24 | 12,697,015 | HiSeq 150 bp paired | DRR278586 |
| 80 | *Ischnura senegalensis* | Isen23 | AF | Thorax | Mature adult | 2017.07.24 | 12,492,681 | HiSeq 150 bp paired | DRR278587 |
| 81 | *Ischnura senegalensis* | Isen23 | AF | Abdomen | Mature adult | 2017.07.24 | 12,282,217 | HiSeq 150 bp paired | DRR278588 |
| 82 | *Ischnura senegalensis* | Isen23 | AF | Wings | Mature adult | 2017.07.24 | 12,001,401 | HiSeq 150 bp paired | DRR278589 |
| 83 | *Ischnura senegalensis* | Isen24 | F | Epidermis, RNAi region, *Kr-h1* RNAi | Day 3, final instar nymph | 2018.11.19 | 18,204,949 | HiSeq 150 bp paired | DRR278598 |
| 84 | *Ischnura senegalensis* | Isen24 | F | Epidermis, control region, *Kr-h1* RNAi | Day 3, final instar nymph | 2018.11.19 | 10,395,142 | HiSeq 150 bp paired | DRR278599 |
| 85 | *Ischnura senegalensis* | Isen25 | F | Epidermis, RNAi region, *Kr-h1* RNAi | Day 2, final instar nymph | 2018.11.16 | 14,291,842 | HiSeq 150 bp paired | DRR278600 |
| 86 | *Ischnura senegalensis* | Isen25 | F | Epidermis, control region, *Kr-h1* RNAi | Day 2, final instar nymph | 2018.11.16 | 13,205,085 | HiSeq 150 bp paired | DRR278601 |
| 87 | *Ischnura senegalensis* | Isen26 | N | Epidermis, RNAi region, *Kr-h1* RNAi | Day 2, penultimate instar nymph | 2017.11.22 | 10,474,065 | HiSeq 150 bp paired | DRR278602 |
| 88 | *Ischnura senegalensis* | Isen26 | N | Epidermis, control region, *Kr-h1* RNAi | Day 2, penultimate instar nymph | 2017.11.22 | 11,669,201 | HiSeq 150 bp paired | DRR278603 |
| 89 | *Ischnura senegalensis* | Isen27 | AF | Epidermis, RNAi region, *E93* RNAi | Day 2, immature adult | 2018.11.28 | 10,882,090 | HiSeq 150 bp paired | DRR278604 |
| 90 | *Ischnura senegalensis* | Isen27 | AF | Epidermis, control region, *E93* RNAi | Day 2, immature adult | 2018.11.28 | 8,968,025 | HiSeq 150 bp paired | DRR278605 |
| 91 | *Ischnura senegalensis* | Isen28 | M | Epidermis, RNAi region, *E93* RNAi | Day 1, immature adult | 2018.11.29 | 14,618,655 | HiSeq 150 bp paired | DRR278606 |
| 92 | *Ischnura senegalensis* | Isen28 | M | Epidermis, control region, *E93* RNAi | Day 1, immature adult | 2018.11.29 | 9,920,849 | HiSeq 150 bp paired | DRR278607 |
| 93 | *Ischnura senegalensis* | Isen29 | M | Epidermis, RNAi region, *E93* RNAi | Day 1, immature adult | 2018.11.29 | 17,515,508 | HiSeq 150 bp paired | DRR278608 |
| 94 | *Ischnura senegalensis* | Isen29 | M | Epidermis, control region, *E93* RNAi | Day 1, immature adult | 2018.11.29 | 9,363,482 | HiSeq 150 bp paired | DRR278609 |
| 95 | *Ischnura senegalensis* | Isen30 | F | Epidermis, RNAi region, *broad* RNAi | Day 2, final instar nymph | 2018.11.19 | 12,463,361 | HiSeq 150 bp paired | DRR278610 |
| 96 | *Ischnura senegalensis* | Isen30 | F | Epidermis, control region, *broad* RNAi | Day 2, final instar nymph | 2018.11.19 | 9,422,215 | HiSeq 150 bp paired | DRR278611 |
| 97 | *Ischnura senegalensis* | Isen31 | M | Epidermis, RNAi region, *broad* RNAi | Day 2, final instar nymph | 2019.04.15 | 10,624,432 | HiSeq 150 bp paired | DRR278612 |
| 98 | *Ischnura senegalensis* | Isen31 | M | Epidermis, control region, *broad* RNAi | Day 2, final instar nymph | 2019.04.15 | 9,927,679 | HiSeq 150 bp paired | DRR278613 |
| 99 | *Ischnura senegalensis* | Isen32 | F | Epidermis, RNAi region, *broad* RNAi | Day 2, final instar nymph | 2019.04.21 | 13,789,904 | HiSeq 150 bp paired | DRR278614 |
| 100 | *Ischnura senegalensis* | Isen32 | F | Epidermis, control region, *broad* RNAi | Day 2, final instar nymph | 2019.04.21 | 8,740,935 | HiSeq 150 bp paired | DRR278615 |
| 101 | *Ischnura senegalensis* | Isen33 | M | Epidermis, RNAi region, *broad* RNAi | Day 2, final instar nymph | 2019.04.28 | 10,048,200 | HiSeq 150 bp paired | DRR278616 |
| 102 | *Ischnura senegalensis* | Isen33 | M | Epidermis, control region, *broad* RNAi | Day 2, final instar nymph | 2019.04.28 | 9,460,555 | HiSeq 150 bp paired | DRR278617 |
| 103 | *Ischnura senegalensis* | Isen34 | M | Epidermis, Abdomen | Day 2, final instar nymph | 2019.04.18 | 10,726,652 | HiSeq 150 bp paired | DRR278590 |
| 104 | *Ischnura senegalensis* | Isen35 | F | Epidermis, Abdomen | Day 2, final instar nymph | 2018.11.21 | 8,746,286 | HiSeq 150 bp paired | DRR278591 |

| | | | | | | | | | |
|---|---|---|---|---|---|---|---|---|---|
| 105 | *Ischnura senegalensis* | Isen36 | F | Epidermis, Abdomen | Day 2, final instar nymph | 2019.04.14 | 9,722,988 | HiSeq 150 bp paired | DRR278592 |
| 106 | *Ischnura senegalensis* | Isen37 | F | Epidermis, Abdomen | Day 2, final instar nymph | 2019.04.15 | 11,410,936 | HiSeq 150 bp paired | DRR278593 |
| 107 | *Ischnura senegalensis* | Isen38 | M | Epidermis, Abdomen | Day 2, final instar nymph | 2018.11.26 | 11,570,925 | HiSeq 150 bp paired | DRR300699 |
| 108 | *Ischnura senegalensis* | Isen39 | M | Epidermis, Abdomen | Day 2, immature adult | 2018.11.28 | 10,321,054 | HiSeq 150 bp paired | DRR278594 |
| 109 | *Ischnura senegalensis* | Isen40 | GF | Epidermis, Abdomen | Day 2, immature adult | 2020.05.30 | 7,551,012 | HiSeq 150 bp paired | DRR278595 |
| 110 | *Ischnura senegalensis* | Isen41 | GF | Epidermis, Abdomen | Day 2, immature adult | 2020.06.24 | 7,069,194 | HiSeq 150 bp paired | DRR278596 |
| 111 | *Ischnura senegalensis* | Isen42 | AF | Epidermis, Abdomen | Day 2, immature adult | 2018.11.26 | 8,565,695 | HiSeq 150 bp paired | DRR278597 |
| 112 | *Ischnura senegalensis* | Isen43 | GF | Epidermis, Abdomen | Day 2, immature adult | 2018.11.25 | 11,130,097 | HiSeq 150 bp paired | DRR300700 |
| 113 | *Ischnura senegalensis* | Isen44 | M | Epidermis, Abdomen | Day 1, immature adult | 2019.05.22 | 10,224,072 | HiSeq 150 bp paired | DRR300701 |
| 114 | *Ischnura senegalensis* | Isen45 | M | Epidermis, Abdomen | Day 1, immature adult | 2019.05.22 | 7,060,038 | HiSeq 150 bp paired | DRR300702 |
| 115 | *Pseudothemis zonata* | Pzon1 | U | Head | antepenultimate instar nymph | 2018.07.18 | 11,606,250 | HiSeq 150 bp paired | DRR278618 |
| 116 | *Pseudothemis zonata* | Pzon1 | U | Abdomen | antepenultimate instar nymph | 2018.07.18 | 7,336,735 | HiSeq 150 bp paired | DRR278619 |
| 117 | *Pseudothemis zonata* | Pzon2 | U | Head | Early, penultimate instar nymph | 2018.07.18 | 11,696,484 | HiSeq 150 bp paired | DRR278620 |
| 118 | *Pseudothemis zonata* | Pzon2 | U | Abdomen | Early, penultimate instar nymph | 2018.07.18 | 10,998,003 | HiSeq 150 bp paired | DRR278621 |
| 119 | *Pseudothemis zonata* | Pzon3 | U | Head | Late, penultimate instar nymph | 2018.07.05 | 11,491,338 | HiSeq 150 bp paired | DRR278622 |
| 120 | *Pseudothemis zonata* | Pzon3 | U | Abdomen | Late, penultimate instar nymph | 2018.07.05 | 9,656,563 | HiSeq 150 bp paired | DRR278623 |
| 121 | *Pseudothemis zonata* | Pzon4 | U | Head | Stage 1, final instar nymph | 2018.06.23 | 10,069,907 | HiSeq 150 bp paired | DRR278624 |
| 122 | *Pseudothemis zonata* | Pzon4 | U | Abdomen | Stage 1, final instar nymph | 2018.06.23 | 10,000,667 | HiSeq 150 bp paired | DRR278625 |
| 123 | *Pseudothemis zonata* | Pzon5 | M | Head | Stage 2, final instar nymph | 2019.06.17 | 9,418,295 | HiSeq 150 bp paired | DRR278626 |
| 124 | *Pseudothemis zonata* | Pzon5 | M | Abdomen | Stage 2, final instar nymph | 2019.06.17 | 9,959,349 | HiSeq 150 bp paired | DRR278627 |
| 125 | *Pseudothemis zonata* | Pzon6 | F | Head | Stage 2, final instar nymph | 2018.07.18 | 7,898,988 | HiSeq 150 bp paired | DRR278628 |
| 126 | *Pseudothemis zonata* | Pzon6 | F | Abdomen | Stage 2, final instar nymph | 2018.07.18 | 7,492,421 | HiSeq 150 bp paired | DRR278629 |
| 127 | *Pseudothemis zonata* | Pzon7 | M | Head | Stage 3, final instar nymph | 2019.06.17 | 8,770,163 | HiSeq 150 bp paired | DRR278630 |
| 128 | *Pseudothemis zonata* | Pzon7 | M | Abdomen | Stage 3, final instar nymph | 2019.06.17 | 10,081,610 | HiSeq 150 bp paired | DRR278631 |
| 129 | *Pseudothemis zonata* | Pzon8 | F | Head | Stage 3, final instar nymph | 2018.06.23 | 12,504,444 | HiSeq 150 bp paired | DRR278632 |
| 130 | *Pseudothemis zonata* | Pzon8 | F | Abdomen | Stage 3, final instar nymph | 2018.06.23 | 10,387,022 | HiSeq 150 bp paired | DRR278633 |
| 131 | *Pseudothemis zonata* | Pzon9 | M | Head | Day 1, immature adult | 2018.06.22 | 12,757,252 | HiSeq 150 bp paired | DRR278634 |
| 132 | *Pseudothemis zonata* | Pzon9 | M | Abdomen | Day 1, immature adult | 2018.06.22 | 9,287,238 | HiSeq 150 bp paired | DRR278635 |
| 133 | *Pseudothemis zonata* | Pzon10 | F | Head | Day 1, immature adult | 2018.06.22 | 11,078,728 | HiSeq 150 bp paired | DRR278636 |
| 134 | *Pseudothemis zonata* | Pzon10 | F | Abdomen | Day 1, immature adult | 2018.06.22 | 9,501,004 | HiSeq 150 bp paired | DRR278637 |
| 135 | *Pseudothemis zonata* | Pzon11 | M | Head | Mature adult | 2018.07.19 | 8,594,687 | HiSeq 150 bp paired | DRR278638 |
| 136 | *Pseudothemis zonata* | Pzon11 | M | Abdomen | Mature adult | 2018.07.19 | 11,269,401 | HiSeq 150 bp paired | DRR278639 |
| 137 | *Pseudothemis zonata* | Pzon12 | F | Head | Mature adult | 2018.07.19 | 9,324,165 | HiSeq 150 bp paired | DRR278640 |
| 138 | *Pseudothemis zonata* | Pzon12 | F | Abdomen | Mature adult | 2018.07.19 | 8,585,507 | HiSeq 150 bp paired | DRR278641 |

**Table S2.** Summarized results of RNAi experiments in this study.

| Primer No. | Species | Target gene | RNAi region | Injected instar | Observed instar | Injected nymphs | Molted individuals | Individuals with phenotypic effects |
|---|---|---|---|---|---|---|---|---|
| 1 | *Ischnura senegalensis* | N1 | Left abdomen | Penultimate instar nymphs | Final instar nymphs | 16 | 3♂5♀ | 0♂0♀ |
| 2 | *Ischnura senegalensis* | N2 | Left abdomen | Penultimate instar nymphs | Final instar nymphs | 12 | 3♂7♀ | 0♂0♀ |
| 3 | *Ischnura senegalensis* | N3 | Left abdomen | Penultimate instar nymphs | Final instar nymphs | 23 | 2♂5♀ | 0♂0♀ |
| 4 | *Ischnura senegalensis* | N4 (*Kr-h1*) | Left abdomen | Penultimate instar nymphs | Final instar nymphs | 39 | 7♂10♀ | 7♂9♀ |
| | | | Left abdomen | Antepenultimate instar nymphs | Penultimate instar nymphs | 28 | 6♂2♀ | 5♂ |
| | | | Left hindwing (25V) | Penultimate instar nymphs | Final instar nymphs | 4 | 2♂1♀ | 2♂1♀ |
| | | | Left hindwing (10V) | Penultimate instar nymphs | Final instar nymphs | 6 | 3♂3♀ | 0♂0♀ |
| | | | Left abdomen | Final instar nymphs | Emerged adults | 11 | 3♂3A♀2G♀ | 0♂0♀ |
| 5 | *Ischnura senegalensis* | N5 | Left abdomen | Penultimate instar nymphs | Final instar nymphs | 10 | 4♂3♀ | 0♂0♀ |
| 6 | *Ischnura senegalensis* | N6 | Left abdomen | Penultimate instar nymphs | Final instar nymphs | 20 | 2♂6♀ | 0♂0♀ |
| 7 | *Ischnura senegalensis* | N7 (*broad* BTB domain) | Left abdomen | Penultimate instar nymphs | Final instar nymphs | 18 | 6♂8♀ | 6♂7♀ |
| 8 | *Ischnura senegalensis* | N8 | Left abdomen | Penultimate instar nymphs | Final instar nymphs | 9 | 2♂5♀ | 0♂0♀ |
| 9 | *Ischnura senegalensis* | A1 | Left abdomen | Final instar nymphs | Emerged adults | 18 | 8♂4A♀ | 0♂0♀ |
| 10 | *Ischnura senegalensis* | A2 | Left abdomen | Final instar nymphs | Emerged adults | 10 | 4♂3A♀2G♀ | 0♂0♀ |
| 11 | *Ischnura senegalensis* | A3 (*E93*) | Left abdomen | Penultimate instar nymphs | Final instar nymphs and emerged adults | 8 | 4♂1A♀1G♀ | 4♂1A♀1G♀ |
| | | | Left abdomen | Final instar nymphs | Emerged adults | 24 | 6♂3A♀4G♀ | 6♂3A♀4G♀ |
| | | | Left thorax | Final instar nymphs | Emerged adults | 8 | 4♂1A♀3G♀ | 4♂1A♀3G♀ |
| 12 | *Ischnura senegalensis* | A4 | Left abdomen | Final instar nymphs | Emerged adults | 16 | 4♂2G♀ | 0♂0♀ |
| 13 | *Ischnura senegalensis* | A5 | Left abdomen | Final instar nymphs | Emerged adults | 14 | 2♂2A♀2G♀ | 0♂0♀ |
| 14 | *Ischnura senegalensis* | A6 | Left abdomen | Final instar nymphs | Emerged adults | 13 | 5♂1G♀ | 0♂0♀ |
| 15 | *Ischnura senegalensis* | A7 | Left abdomen | Final instar nymphs | Emerged adults | 18 | 4♂4A♀ | 0♂0♀ |
| 16 | *Ischnura senegalensis* | *Met* | Left abdomen | Penultimate instar nymphs | Final instar nymphs | 34 | 11♂3♀ | 10♂3♀ |
| | | | Left abdomen | Final instar nymphs | Emerged adults | 9 | 3♂2A♀3G♀ | 0♂0♀ |
| 17 | *Ischnura senegalensis* | *tai* | Left abdomen | Penultimate instar nymphs | Final instar nymphs | 22 | 4♂6♀ | 4♂6♀ |
| | | | Left abdomen | Final instar nymphs | Emerged adults | 15 | 4♂2A♀4G♀ | 2♂2A♀2G♀ |
| 18 | *Ischnura senegalensis* | *broad* (linker region) | Left abdomen | Penultimate instar nymphs | Final instar nymphs | 53 | 12♂19♀ | 11♂16♀ |
| | | | Left abdomen | Final instar nymphs | Emerged adults | 14 | 4♂3A♀1G♀ | 0♂0♀ |
| | | | Left hindwing (25V) | Penultimate instar nymphs | Final instar nymphs | 2 | 1♂1♀ | 1♂1♀ |
| | | | Left hindwing (10V) | Penultimate instar nymphs | Final instar nymphs | 6 | 4♂2♀ | 0♂0♀ |
| 22 | *Ischnura senegalensis* | *broad* (ZFd) | Left abdomen | Penultimate instar nymphs | Final instar nymphs | 10 | 2♂5♀ | 0♂0♀ |
| 23 | *Ischnura senegalensis* | *broad* (ZFe) | Left abdomen | Penultimate instar nymphs | Final instar nymphs | 10 | 4♂1♀ | 0♂0♀ |
| 24 | *Ischnura senegalensis* | *broad* (ZFf) | Left abdomen | Penultimate instar nymphs | Final instar nymphs | 10 | 5♂2♀ | 0♂0♀ |
| 22,23 | | ZFd and ZFe | Left abdomen | Penultimate instar nymphs | Final instar nymphs | 16 | 7♀ | 4♀ |
| 19-25 | | Mixture of ZFa-ZFg | Left abdomen | Penultimate instar nymphs | Final instar nymphs | 16 | 3♂6♀ | 3♂4♀ |
| 26 | *Pseudothemis zonata* | *Kr-h1* | Right abdomen | Antepenultimate instar nymphs | Penultimate instar nymphs | 31 | 13 | 10 |
| | | | Right abdomen | Penultimate instar nymphs | Final instar nymphs | 58 | 28 | 18 |
| 27 | *Pseudothemis zonata* | *Met* | Right abdomen | Antepenultimate instar nymphs | Penultimate instar nymphs | 15 | 5 | 5 |
| | | | Right abdomen | Penultimate instar nymphs | Final instar nymphs | 42 | 28 | 24 |
| 28 | *Pseudothemis zonata* | *broad* (BTB domain) | Right abdomen | Antepenultimate instar nymphs | Penultimate instar nymphs | 18 | 3 | 2 |
| | | | Right abdomen | Penultimate instar nymphs | Final instar nymphs | 52 | 27 | 2 |
| 29 | *Pseudothemis zonata* | *E93* | Right abdomen | Final instar nymphs | Emerged adults | 12 | 3♂5♀ | 3♂5♀ |
| 30 | *Ischnura senegalensis* | *bla* (negative control) | Left abdomen | Penultimate instar nymphs | Final instar nymphs | 23 | 3♂10♀ | 0 |
| | | | Left abdomen | Final instar nymphs | Emerged adults | 27 | 9♂6A♀6G♀ | 0 |
| | | | Left hindwing (25V) | Penultimate instar nymphs | Final instar nymphs | 6 | 2♂4♀ | 2♂4♀ |
| | | | Left hindwing (10V) | Penultimate instar nymphs | Final instar nymphs | 9 | 4♂5♀ | 1♀ |
| | *Pseudothemis zonata* | | Right abdomen | Final instar nymphs | Emerged adults | 10 | 4 | 0 |
| | | | Right abdomen | Antepenultimate instar nymphs | Penultimate instar nymphs | 19 | 8 | 0 |
| | | | Right abdomen | Penultimate instar nymphs | Final instar nymphs | 35 | 12 | 0 |

**Table S3.** Primers used for dsRNA synthesis in this study.

| | Species | Target gene | Forward primer | Reverse primer |
|---|---|---|---|---|
| 1 | *Ischnura senegalensis* | N1 | TGCCTGTCTCTCATCGTCTG | CGTAGTTGTCCTCGTCGTCA |
| 2 | *Ischnura senegalensis* | N2 | CTCCACAACTTCGCTCACAA | CGTGGTCCTCCTGTGGTAAT |
| 3 | *Ischnura senegalensis* | N3 | GCAACGACTTCAACCCAAAT | ACAAAGAGTGGGCGTGAAAC |
| 4 | *Ischnura senegalensis* | N4 (*Kr-h1*) | AACCGTACCAATGCGAGTTC | GAGCTTGAGGACGTGGTTGT |
| 5 | *Ischnura senegalensis* | N5 | TCACCCATGCATTGTTTGAG | CCACAAGAGGTGATTGGACA |
| 6 | *Ischnura senegalensis* | N6 | CTTGCGTCTTTTCCAAAGGT | GGCAACTTCTTCCAGCAGAC |
| 7 | *Ischnura senegalensis* | N7 (*broad* BTB domain) | CCCATAGGGTTGTGCTGTCT | TTTGCCGTTCTCCTCTGAGT |
| 8 | *Ischnura senegalensis* | N8 | GGTAAATCAAGGTTGATCAAGGTAA | ACCGAACTGGCAAGTCCTC |
| 9 | *Ischnura senegalensis* | A1 | CCATAAACCCAAATCCATCG | ACCACGCGAGTATTGGAGAC |
| 10 | *Ischnura senegalensis* | A2 | AGAGGTTCGAACACCGCTAA | CGGAATACCAAGCTACATCCA |
| 11 | *Ischnura senegalensis* | A3 (*E93*) | CACAGGAGATGCACATGGAC | CAGTCATTCCTCGACCACCT |
| 12 | *Ischnura senegalensis* | A4 | GTTGGCTGCTTGATGGATTT | TTCGACATGGTGCACGTTAG |
| 13 | *Ischnura senegalensis* | A5 | ACAATGACGCCAAGGAAAAC | TCGAAGACGTCGCTCTGATA |
| 14 | *Ischnura senegalensis* | A6 | CTGGAGGTGTTCATCGAGGT | GGACCTGGACCTTTCCATCT |
| 15 | *Ischnura senegalensis* | A7 | GGGCTTCATTTCCTGGACTA | GGGGTAGTTTTCACCCCTAAA |
| 16 | *Ischnura senegalensis* | *Met* | TGATGGTGCCAATTCAAAAA | CATGTAACCAGCCACCACAG |
| 17 | *Ischnura senegalensis* | *tai* | TGAGCAGGATTTCATCATGG | GAGGAGGTGGGGAAGAAGTC |
| 18 | *Ischnura senegalensis* | *broad* (linker region) | ACTCAGAGGAGAACGGCAAA | TGTCATCGTCCTCATCTTCG |
| 19 | *Ischnura senegalensis* | *broad* (ZFa) | TGCGACAGGAAGAGCATACA | CTTCCTCGCATACGGACAC |
| 20 | *Ischnura senegalensis* | *broad* (ZFb) | TTTCTTGCAGCAGAGGAGTG | TGAGAAATGAGGGAGCTTCG |
| 21 | *Ischnura senegalensis* | *broad* (ZFc) | CTCGTCCCCTCCCAACAC | CCCGGTGATAGATGCTCTTG |
| 22 | *Ischnura senegalensis* | *broad* (ZFd) | AATCCTGGCACCCCATTTA | GTGGTTGTGCAGGCTGTTTA |
| 23 | *Ischnura senegalensis* | *broad* (ZFe) | GCCCCTCATCGGCTACTG | CCGGCTCTTATGGTAGGTGT |
| 24 | *Ischnura senegalensis* | *broad* (ZFf) | GTTGAGTCCCAATGGCAGAT | ATTTACTCCTCCGTCGCAGA |
| 25 | *Ischnura senegalensis* | *broad* (ZFg) | CAGGAGCTTGTTTGCCATTT | AAGAACTGCACGGAGAGGAA |
| 26 | *Pseudothemis zonata* | *Kr-h1* | GCTCACTTTGGGGAGAAGGT | TGGACAGTTTCTGTTCTCCTTG |
| 27 | *Pseudothemis zonata* | *Met* | CAAGTGGAGAGGCTTCTTGG | TTTGCGACTCTTCAGTGGTG |
| 28 | *Pseudothemis zonata* | *broad* (BTB domain) | GACCTGCATGCTCTTGTTGA | CGGAACTCCTGAGTGGTCAT |
| 29 | *Pseudothemis zonata* | *E93* | TCTCCATCCATCACCCTCTC | CCGTTCCATGGCTAACTTGT |
| 30 | | *bla* (negative control) | CTATGTGGCGCGGTATTAT | CAGAAGTGGTCCTGCAACT |

**Dataset S1 (separate file).** Nucleotide and amino acid sequences, annotation result, and raw expression data (TPM values) of adult-epidermis-specific (AES) genes and nymphal-epidermis-specific (NES) genes.

**SI References**


1. R. J. Tillyard, *The biology of dragonflies* (Cambridge University Press, 1917).
2. P. S. Corbet, *Dragonflies, Behavior and Ecology of Odonata* (Cornell University Press, 1999).
3. G. Okude, R. Futahashi, M. Tanahashi, T. Fukatsu, Laboratory rearing system for *Ischnura senegalensis* (Insecta: Odonata) enables detailed description of larval development and morphogenesis in dragonfly. *Zoolog Sci* **34**, 386–397 (2017).
4. G. Okude, T. Fukatsu, R. Futahashi, Electroporation-mediated RNA interference method in Odonata. *J Vis Exp* **168**, e61952 (2021).
5. A. Ozono, I. Kawashima, R. Futahashi R, *The Handbook of Japanese Aquatic Insects. Volume 3: Dragonfly larvae* (Bunichi-Sogo Syuppan Co Ltd, 2019).
6. G. Okude, T. Fukatsu, R. Futahashi, Comprehensive comparative morphology and developmental staging of final instar larvae toward metamorphosis in the insect order Odonata. *Sci Rep* **11**, 5124 (2021).
7. S. Andrews, FastQC: A Quality Control Tool for High Throughput Sequence Data [Online]. (2010) Available from URL: http://www.bioinformatics.babraham.ac.uk/projects/fastqc/
8. A. M. Bolger, M. Lohse, B. Usadel, Trimmomatic: a flexible trimmer for Illumina sequence data. *Bioinformatics* **30**, 2114–2120 (2014).
9. M. G. Grabherr *et al.*, Full-length transcriptome assembly from RNA-Seq data without a reference genome. *Nat Biotechnol* **29**, 644–652 (2011).
10. J. T. Robinson *et al.*, Integrative Genomics Viewer. *Nat Biotechnol* **29**, 24–26 (2011).
11. R. Patro, G. Duggal, M. I. Love, R. A. Irizarry, C. Kingsford, Salmon provides fast and bias-aware quantification of transcript expression. *Nat Methods* **14**, 417–419 (2017).
12. M. D. Robinson, D. J. McCarthy, G. K. Smyth, edgeR: a Bioconductor package for differential expression analysis of digital gene expression data. *Bioinformatics* **26**, 139–140 (2010).
13. S. F. Altschul *et al.*, Gapped BLAST and PSI-BLAST: a new generation of protein database search programs. *Nucleic Acids Res* **25**, 3389–3402 (1997).
14. J. J. A. Armenteros *et al.*, SignalP 5.0 improves signal peptide predictions using deep neural networks. *Nat Biotechnol* **37**, 420-423 (2019).
15. R Core Team, R: A language and environment for statistical computing. (R Foundation for Statistical Computing, 2018). Available from URL: https://www.R-project.org/
16. J. H. Willis, Structural cuticular proteins from arthropods: annotation, nomenclature, and sequence characteristics in the genomics era. *Insect Biochem Mol Biol* **40**, 189-204 (2010).
17. A. Untergasser *et al*., Primer3--new capabilities and interfaces. Nucleic Acids Res **40**, e115 (2012).
18. R. Futahashi, Whole-mount *in situ* hybridization of sectioned tissues of species hybrids to detect *cis*-regulatory changes in gene expression pattern. *Methods Mol Biol* **772**, 319–328 (2011).
19. Y. Matsuura, Y. Kikuchi, T. Miura, T. Fukatsu, Ultrabithorax is essential for bacteriocyte development. *Proc Natl Acad Sci USA* **112**, 9376–9381 (2015).
20. S. Kumar, G. Stecher, K. Tamura, MEGA7: Molecular evolutionary genetics analysis version 7.0 for bigger datasets. *Mol Biol Evol* **33**: 1870–1874 (2016).
21. I. Almudi *et al.*, Genomic adaptations to aquatic and aerial life in mayflies and the origin of insect wings. *Nat Commun* **11**, 2631 (2020).
22. R. Futahashi, M. Osanai-Futahashi, “Pigments in insects” in Pigments, *Pigment Cells and Pigment Patterns*, H. Hashimoto, M. Goda, R. Futahashi, R. Kelsh, T. Akiyama, Eds (Springer, 2021), pp. 3–43.
23. E. Ureña, C. Manjon, X. Franch-Marro, D. Martin, Transcription factor E93 specifies adult metamorphosis in hemimetabolous and holometabolous insects. *Proc Natl Acad Sci USA* **111**, 7024–7029 (2014).
24. Y. Ishimaru, S. Tomonari, T. Watanabe, S. Noji, T. Mito, Regulatory mechanisms underlying the specification of the pupal-homologous stage in a hemimetabolous insect. *Philos Trans R Soc Lond B Biol Sci* **374**, 20190225 (2019).

25. X. Belles, *Insect metamorphosis: From natural history to regulation of development and evolution* (Academic Press, 2020).
26. C. Minakuchi, X. Zhou, L. M. Riddiford, *Krüppel homolog 1* (*Kr-h1*) mediates juvenile hormone action during metamorphosis of *Drosophila melanogaster*. *Mech Dev* **125**: 91–105 (2008).
